# Development of a Wine By‐Product‐Based Beverage and Study of Its Potential to Postprandial Glycemia Regulation in Healthy Individuals: A Proof of Concept Study

**DOI:** 10.1002/mnfr.70128

**Published:** 2025-05-27

**Authors:** María‐José Motilva, Silvia Yuste, Juana I. Mosele

**Affiliations:** ^1^ Instituto de Ciencias de la Vid y del Vino‐ICVV (Consejo Superior de Investigaciones Científicas‐CSIC Universidad de La Rioja‐UR, Gobierno de La Rioja), Finca La Grajera Logroño Spain; ^2^ Antioxidants Research Group, Food Technology Department, Agrotecnio‐RECERCA Center University of Lleida Lleida Spain; ^3^ Faculty of Health Sciences Universidad de La Rioja Logroño Spain; ^4^ Fisicoquímica, Facultad de Farmacia y Bioquímica‐IBIMOL Universidad de Buenos Aires‐CONICET Buenos Aires Argentina

**Keywords:** grape functional ingredients, phenol metabolites, postprandial glycaemia, UPLC‐MS/MS, wine by‐products, wine by‐product beverage

## Abstract

Aligned with the principles of the circular bioeconomy, this study explores the use of winemaking by‐products as a source of bioactive phenolic compounds. Skins and seeds from Tempranillo and Graciano grapevine varieties were conditioned to prepare a phenolic‐rich beverage. To study its hypoglycemic properties, a pilot trial was conducted to evaluate postprandial glycemia after the intake of the wine by‐product‐based beverage (WBB). The postprandial glycemia of 10 healthy adults was measured after the intake of: (i) water + sugar solution, (ii) WBB + sugar solution, and (iii) WBB + water. In parallel to blood glucose measurement, blood samples were also collected using dried blood spot cards to determine the phenolic metabolites by UHPLC‐QqQ‐MS/MS. Although blood glucose levels tended to decrease after WBB intervention, these changes were not statistically significant related to the interindividual variability. The volunteers showing a reduction in blood glucose were classified as “responders”, and those who showed no changes were classified as “non‐responders”. Wine by‐products can be effectively used in functional beverages formulation to increase bioactive compound content and manage postprandial glucose in some individuals.

## Graphical abstract

The postprandial glycemia of healthy adults is not downregulated by the coadministration of a sugar solution and a wine by‐product enriched beverage due to the high variability in the response. Blood phenolic compounds of responders’ volunteers are not associated with the reduction effect of postprandial hyperglycemia.

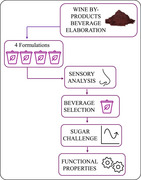

## 1 Introduction

The recovery of the agri‐food by‐products, derived from vegetable processing, for revaluation as functional food ingredients aligns with the principles of the circular bioeconomy. Sustainable functional foods are highly valued for their lower environmental impact and enhanced health benefits compared to conventional foods [1, 2]. By‐products generated from plant processing retain considerable amounts of the bioactive compounds from the original raw materials, making them a valuable source of ingredients for the development of functional foods [1, 2, 3, 4]. A recent publication by our group showed that the winemaking process generates various phenol‐rich solid by‐products. These include stems and grape‐pomace (a mixture of the skins and seeds), with red grape skins being particularly rich in anthocyanins and seeds being an excellent source of noncolored phenolic compounds, especially flavan‐3‐ols [5].

Wine by‐products can be used directly in food formulation as ingredients following minimal processing, such as drying, milling, or sieving, or they can be used as extracts after concentrating their bioactive compounds [1, 6]. The former option is more environmentally friendly due to the low or negligible generation of chemical waste. However, it is less popular because it could introduce negative sensory attributes to the final product. The formulation of novel foods using these types of by‐products is expected to open new opportunities in areas such as environmental sustainability, reinforcing the application of circular bioeconomy, food innovation, and health by offering natural alternatives for chronic disease prevention [1, 2, 4]. The healthy attributes of agro‐food by‐products have been evidenced in recent studies. For instance, adding grape‐pomace flour to biscuits increases the levels of (poly)phenols and dietary fiber, thereby contributing positively to the daily intake of these bioactive components [1], something which has been associated with a reduced risk of chronic diseases such as diabetes [6, 7, 8].

Chronic diseases are closely linked to lifestyle factors, with diet being a key aspect [9]. Diets high in refined carbohydrates increase the risk of metabolic glucose alterations. For example, frequent consumption of foods with a high glycemic index leads to repeated episodes of postprandial hyperglycemia, increasing the likelihood of a cascade of metabolic dysfunctions that can eventually progress to Type‐2 diabetes [9]. Therefore, reducing high blood glucose levels by managing glucose absorption/metabolism during the digestion process helps to prevent the premature onset of metabolic disorders associated with postprandial glucose management. In this context, maintaining glucose homeostasis is crucial to reducing the risk of diabetes due to postprandial hyperglycemia. Furthermore, long‐term exposure to high blood glucose levels can cause oxidative stress and inflammation, further contributing to the development of metabolic disorders [3, 9]. Therefore, managing glucose levels during the postprandial period may reduce the risk of developing further metabolic issues.

The antidiabetic properties of wine byproducts, particularly grape‐pomace, have been investigated in previous studies [1, 6]. Different mechanisms of glucose regulation by grape‐pomace (poly)phenols have been proposed, including the reduction of glucose intestinal absorption, inhibition of digestive enzyme activity, modulation of glucose release from the liver, enhancement of insulin sensitivity, stimulation of insulin secretion, modulation of intracellular signaling pathways, and gene expression [3, 6]. However, none of these studies have considered the analysis of blood phenolic metabolites during the postprandial state in humans to determine their potential role in the management of glucose metabolism.

To the best of our knowledge, no previous works have explored the effectiveness of wine by‐products as functional ingredients for blood glucose control during the postprandial state in humans. Considering this gap, we propose to explore the functionality of a wine by‐product‐based beverage (WBB) rich in highly diversified (poly)phenolic compounds to control postprandial blood glucose levels after an acute intake of a sugar solution in healthy individuals. In parallel, the concentration of phenol biological metabolites in blood samples during the postprandial period was determined to assess their potential role in regulating the postprandial glycemia. The presence of WBB phenolic metabolites in human blood during the postprandial period may be involved in the modulation of the metabolic pathways responsible for glucose metabolism regulation after high sugar intake.

## 2 Material and Methods

### 2.1 Chemical and Reagents

Methanol (HPLC grade), formic acid (HPLC grade), acetonitrile (HPLC grade), and HCl were purchased from VWR Chemicals BDH Prolabo (Leuven, Belgium). The water was Milli‐Q quality (Millipore Corp, Bedford, MA, USA). The commercial standards of quercetin, quercetin‐3‐glucuronide, *trans‐*resveratrol, *trans‐*resveratrol‐glucoside, (−)‐epicatechin, dimers B1 and B2, and 3‐*O*‐glucosides of cyanidin, delphinidin, malvidin, peonidin, petunidin, isorhamnetin, and syringetin were purchased from Extrasynthese (Genay, France). (+)‐Catechin, *p*‐hydroxybenzoic acid, 3,4‐dihydroxybenzoic acid (protocatechuic acid), *p*‐coumaric acid, gallic acid, caffeic acid, ferulic acid, vanillic acid, syringic acid, matairesinol, and secoisolariciresinol were acquired from Sigma–Aldrich (St. Louis, USA). Caftaric acid and kaempferol‐3‐glucoside were purchased from Purifa‐Cymit (Barcelona, Spain). Naringerin and coutaric acid were purchased from Fluochem (Hadfield, UK) and Phytolab (Madrid, Spain), respectively. 4,4‐*Bis*(4‐hydroxyphenyl)valeric acid (Sigma–Aldrich), 5‐(3′,4′‐dihydroxyphenyl)‐δ‐valerolactone (TransMIT, Gießen, Germany), 3‐(3,4‐dihydroxyphenyl)propionic acid and 3,4‐dihydroxyphenylacetic acid (Alfa Aesar, MA, USA), 3‐(3‐hydroxyphenyl)propionic acid (Biosynth Carbosynth, Compton, UK), hippuric acid (HA) and 3‐phenylpropionic acid (Thermo Fisher Scientific, Walthman, MA, USA), catechol (TCI, Tokio, Japan), pyrogallol (Glentham Life Sciences, Corsham, UK), and gallocatechin (Target Mol, MA, USA). Stock solutions of each standard were prepared in methanol (1000 mg/L) and stored at −20°C.

### 2.2 Elaboration and Analysis of Wine By‐Product‐Based Beverage

The wine by‐products (skins and seeds) used as ingredients for the elaboration of the WBB were obtained from lyophilized grape pomace of Tempranillo and Graciano red grape cultivars, characteristic of the La Rioja wine region. The wine by‐products (2021 harvest) were collected in the experimental winery of the Instituto de Ciencias de la Vid y el Vino (ICVV, La Rioja, Logroño, Spain). The selection of these grape varieties due to the possibility of the residual presence of alcohol, the grape pomace samples were freeze‐dried in a Lyophilizer Telstar LyoQuest‐85 (Terrassa, Spain). After lyophilization, the dehydrated grape pomace was sieved to give two fractions: skin and seeds. All dehydrated samples (skin and seeds) were ground (IKA basic analytical mill, Staufen, Germany), sieved (*ø* 0.5 mm), and stored at −80°C until their use for WBB formulation.

Four formulations of WBB were explored to achieve an optimal balance between sensory acceptability and the maximization of (poly)phenol concentration. The WBBs were prepared using a fixed amount of natural mineral water, thickener (Resource Clear, Nestle Health Science), and different percentages of sifted lyophilized skins and seeds (Table 1). Each WBB formula was prepared by weighing the dry ingredients and mixing them with water through vigorous manual agitation before the WBB intake. The addition of the thickener helped to stabilize the beverage by slowing the separation of skins and seeds (by‐products) from the aqueous phase.

**TABLE 1:** Formulations of wine byproducts‐based beverages (WBB).

Formulation WBB	1	2	3	4
Tempranillo skin (%)	2.5	5.0	2.0	4.0
Graciano skin (%)	2.5	5.0	2.0	4.0
Grape seed (%)	−	−	1.0	2.0
Total grape by‐products (%)	5.0	10	5.0	10
Thickener (%)	0.4	0.4	0.4	0.4
Water (%)	94.6	89.6	94.6	89.6
Concentration (mg/200 mL WBB)				
Total malvidins	45.64 ± 2.38^a^	91.29 ± 4.75^c^	36.90 ± 0.01^b^	73.79 ± 0.02^d^
Total petunidins	9.59 ± 0.39^a^	19.19 ± 0.78^b^	7.73 ± 0.002^a^	15.45 ± 0.003^b^
Total delphinidins	15.57 ± 0.45^a^	31.14 ± 0.90^b^	12.52 ± 0.003^a^	25.04 ± 0.01^c^
Total peonidins	8.70 ± 0.59^a^	17.41 ± 1.18^b^	6.98 ± 0.002^a^	13.96 ± 0.005^b^
Total cyanidins	1.72 ± 0.08	3.43 ± 0.17	1.38 ± 0.0004	2.76 ± 0.001
Total Pelargonidins	0.02 ± 0.001	0.03 ± 0.001	0.01 ± 0.000001	0.03 ± 0.00001
Minor compounds (vitisins and pinotin A)	0.51 ± 0.02	1.01 ± 0.03	0.41 ± 0.0002	0.82 ± 0.0004
Total anthocyanins	81.75 ± 3.90^a^	163.49 ± 7.80^c^	65.93 ± 0.02^b^	131.87 ± 0.04^d^
Total hydroxycinnamic acids	1.76 ± ± 0.11	3.52 ± 0.21	1.58 ± 0.003	3.16 ± 0.01
Total hydroxybenzoic acids	2.13 ± 0.07	4.26 ± 0.14	3.13 ± 0.001	6.27 ± 0.001
Total phenolic acids	3.89 ± 0.16^a^	7.78 ± 0.32^ab^	4.72 ± 0.003^a^	9.43 ± 0.01^b^
Total phenyl alcohols	1.11 ± 0.17	2.22 ± 0.35	1.05 ± 0.002	2.09 ± 0.005
Total flavanones	0.04 ± 0.001	0.08 ± 0.003	0.03 ± 0.00002	0.07 ± 0.00004
Total myricetins	2.94 ± 0.18	5.87 ± 0.36	2.37 ± 0.001	4.74 ± 0.003
Total quercetin	10.28 ± 0.49^a^	20.55 ± 0.99^b^	8.38 ± 0.003^a^	16.75 ± 0.01^b^
Total minor compounds	1.36 ± 0.06	2.72 ± 0.12	1.10 ± 0.001	2.21 ± 0.001
Total flavonols	14.57 ± 0.73^a^	29.15 ± 1.46^b^	11.85 ± 0.005^a^	23.70 ± 0.01^c^
Total catechin derivatives	1.84 ± 0.05^a^	3.68 ± 0.10^ab^	3.55 ± 0.001^ab^	7.11 ± 0.001^b^
Total procyanidins	2.95 ± 0.13^a^	5.90 ± 0.26^ab^	4.81 ± 0.002^a^	9.62 ± 0.004^b^
Total flavan‐3‐ols	4.79 ± 0.08^a^	9.58 ± 0.16^ab^	8.36 ± 0.002^ab^	16.72 ± 0.003^c^
Total stilbenes	0.47 ± 0.01^a^	0.94 ± 0.02^a^	0.38 ± 0.00005^a^	0.77 ± 0.0001^a^
Total lignans	0.43 ± 0.02	0.87 ± 0.04	0.70 ± 0.001	1.41 ± 0.001
Total noncolored phenols	25.31 ± 0.98^a^	50.63 ± 1.96^b^	27.10 ± 0.005^a^	54.20 ± 0.01^b^
Total (poly)phenols	**107.06** ± **4.83^a^**	**214.12** ± **9.66^c^**	**93.03** ± **0.021^b^**	**186.06** ± **0.04^d^**
Soluble fiber (pectin)	**265.60** ± **5.60a**	**531.20** ± **11.20c**	**332.00** ± **2.20b**	**664.00** ± **4.40d**

*Note*: Content of (poly)phenols and soluble fiber (pectin) in 200 mL, equivalent to the dose ingested through the acute intake. Different letters denote statistically significant differences among group means based on Tukey's honest significant difference (HSD) test following a one‐way ANOVA (*p* < 0.05).

The method for determining the (poly)phenol composition of skins, seeds, and WBB by liquid chromatography coupled to mass spectrometry (HPLC‐MS/MS) has been reported recently by our group [5]. The (poly)phenol composition of the skin and seeds is available in Table S1. The determination of the pectin (soluble fiber) content in the grape skins was carried out using a gravimetric procedure following the instructions in Mosele et al. [5]. For this, 1 g of skin sample was stirred at 85°C for 40 min with 20 mL of HCl 0.02 M and then filtered. Once the filtrate had cooled to 30°C, ethanol at 60°C (1:4, filtrated/ethanol, v/v) was added and then centrifuged for 30 min at 9000 rpm after resting for 24 h at 4°C. The liquid residue was discarded, and the remaining solid residue was dried at 55°C until a constant weight was achieved. The percentage of pectin in the skins was then calculated considering the dry weight of the skin sample and the dry weight of the extracted solid residue (pectin).

### 2.3 Sensory Evaluation of the WBBs

Consumer acceptance of the four different WBB formulations (Table 1) was assessed by a group of 10 untrained volunteers (aged 26–56 years), consisting of 50% women. Participants were selected based on the absence of alterations in olfactory and taste perception, allergy or intolerance to grapes, as well as not using medication that could affect sensory experience. Only data regarding age and gender was collected as demographic information. The volunteers tested the four WBB formulations on the same day and evaluated such key characteristics as appearance, taste, aroma, mouthfeel (texture), and overall acceptance, as previously assigned by an expert. These five categories, along with their respective descriptors, were presented on a tasting sheet with an intensity line scale (9‐point anchored, nonstructured hedonic scale, Figure SF1) where “no perception” was rated as 1 and “extreme perception” as 9 [10]. The formulations were served at 10°C in a randomized order and identified with internal codes.

The relative frequency (*F*), relative intensity (*I*), and geometric mean (GM) of the different descriptors were calculated for each WBB formulation. The GM was determined as the square root of the product of *I* and *F*, expressed as GM (%) = √(*I* × *F*) × 100. In this context, (*I*) represents the sum of the intensities assigned by the panel for a given descriptor, divided by the maximum possible intensity for that descriptor, while (*F*) corresponds to the number of times the descriptor was mentioned, divided by the maximum number of times it could be mentioned.

### 2.4 Postprandial Blood Glycemia Study

#### 2.4.1 Participants

A human pilot postprandial study was conducted with 10 healthy adults (50% female, 26–46 years, IMC <25 kg/m^2^) recruited from the ICVV. The volunteers reported no history of glucose metabolism disorders (e.g., diabetes, hyperinsulinemia, and prediabetes), no intolerance or allergy to grapes or grape‐based products, and were not pregnant, lactating, or smokers. All participants received both written and oral information about the objectives of the study, and informed consent was obtained from each one to confirm their voluntary participation. Medical staff were present during each visit to assist with blood collection and to support volunteers in case of any adverse effects resulting from fasting, WBB, and/or sugar intake. No adverse effects were reported by any volunteers during any of the interventions.

#### 2.4.2 Human Intervention

**FIGURE 1:**
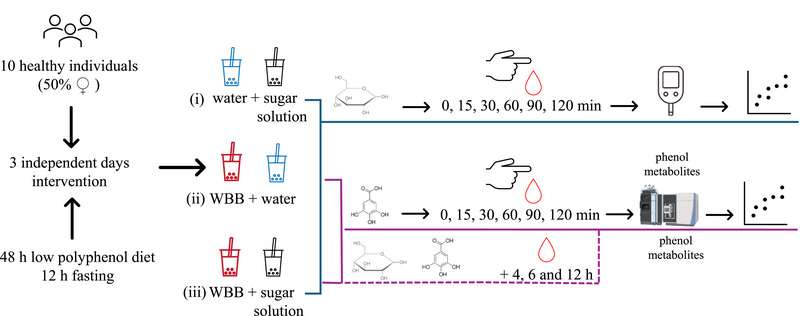
Study design of the postprandial glycemia pilot study (3 independent days of intervention).

The design of the pilot study was approved by the Bioethics and Biosafety Committee of the Consejo Superior de Investigaciones Científicas (CSIC, Madrid, Spain) (internal code 034/2023). The procedure involved a within‐subject intervention conducted over three separate days. During each session, volunteers received one of the three different oral treatments, which each session spaced 7 days apart (Figure 1). On each intervention day, the volunteers arrived at the center after a 12‐h fasting and after following a low (poly)phenol diet for 48 h. To encourage adherence to the low polyphenol diet, a dietitian provided a list of foods categorized into three groups: allowed, allowed in very small amounts, and not allowed. The “not‐allowed” group included all grape‐derived products (red and white wine, juice, and raisins), red berries, coffee, tea, extra virgin olive oil, beer, whole grains, and chocolate. Additionally, volunteers received a list of suggested meals to prepare during the 2‐day restricted diet. On each of the three separate occasions, each participant received different treatments. The intervention involved the acute intake of two different preparations ingested within 5–10 min: (i) water + sugar solution (30 g of available carbohydrates, provided as commercial sucrose dissolved in 50 mL of mineral water); (ii) WBB + water; and (iii) WBB + sugar solution (Figure 1).

#### 2.4.3 Blood Sample Collection

Blood samples for determining glucose and phenolic biological metabolites were obtained via a finger‐pick using a sterile lancet (21G‐18 mm depth, Vitrex Medical, Denmark). To monitor postprandial glycemia, blood glucose was measured at 0, 15, 30, 60, 90, and 120 min after ingestion using a commercial glucometer (Contour XT, Bayer).

For phenol metabolite determination, blood samples were collected directly on FTA DMPK‐A cards (DBS filter paper) (GB Healthcare, Buckinghamshire, UK) until the entire designated circle on the card was filled. For each volunteer, a blood sample was collected at 0, 15, 30, 60, 90, and 120 min after the three interventions, as well as at 4, 6, and 12 h after the volunteers consumed WBB + water (ii) and WBB + sugar solution (iii) (Figure 1). Blood samples corresponding to 12 h after the interventions (ii) and (iii) were self‐collected by volunteers at home following instructions provided. For each sampling, the volunteer filled four circles of the DBS filter paper with the blood sample. The DBS cards were stored in a desiccator in the dark for 15 days until the chromatographic analysis of the phenol biological metabolites. For the concentrations of both glucose and phenolic compounds in the blood, the incremental area under the curve (iAUC) was calculated using the trapezoidal method, ignoring all the area under the baseline.

### 2.5 Analysis of the Blood Phenolic Metabolites by Ultra‐Performance Liquid Chromatography Coupled to Tandem Mass Spectrometry (UHPLC‐QqQ‐MS/MS)

#### 2.5.1 Blood Sample Pretreatment

Blood samples collected in DBS cards were processed according to the methodology described by Yuste et al. [11]. Briefly, two whole circles of DBS cards containing blood samples were perforated using a punch to obtain smaller pieces, which were extracted with 150 µL of MeOH:H_2_O (50:50, v/v). After centrifuging (14,000 rpm, 4°C, 15 min), the supernatant was injected into the chromatographic system. Each blood collection time point (0, 15, 30, 60, 90, and 120 min and 4, 6, and 12 h) was analyzed in duplicate using two circles of DBS filter paper representing a single replicate.

#### 2.5.2 UHPLC‐QqQ‐MS/MS

The phenol metabolites in the blood samples were analyzed by UHPLC‐QqQ‐MS/MS. Liquid chromatography was performed on a Shimadzu Nexera system (Shimadzu Corporation, Japan), coupled to a QTRAP mass spectrometer (AB Sciex 3200QTRAP, Sciex, USA). The separation of the phenol compounds was done on a Waters AcQuity BEH C18 column (100 × 2.1 mm, 1.7 µm particle size; Waters, Milford, MA, USA) with a VanGuardTM AcQuity BEH C18 Pre‐Column (5 × 2.1 mm, 1.7 µm particle size; Waters). Two chromatographic methods were used, one for the analysis of anthocyanin metabolites and another to analyze the other phenolic compounds. Both methods utilized a flow rate of 0.45 mL/min and a sample injection volume of 2.5 µL. The autosampler and oven temperatures were set to 5 and 40°C, respectively. For the separation of anthocyanin metabolites, the mobile phase consisted of 2% formic acid in water (Solvent A) and 2% formic acid in acetonitrile (Solvent B). For the separation of the other phenolic compounds, the mobile phase was 0.1% formic acid in water (Solvent A) and in acetonitrile (Solvent B).

The eluted compounds were analyzed using a triple quadrupole mass spectrometer (qQq‐MS/MS) equipped with an electrospray interface (ESI). The ESI was operated in positive ion mode [M–H]^+^ for the analysis of the anthocyanin metabolites and in negative ion mode [M–H]^−^ for the analysis of other phenol metabolites. Data acquisition was performed using multiple reaction monitoring (MRM), where two MRM transitions were monitored: one for quantification and a second for confirmation. Table 2S provides details on the retention time (*t*_R_) and MRM transitions and parameters for each phenolic compound, including declustering potential (DP), entrance potential (EP), collision cell entrance potential (CEP), collision energy (CE), and collision cell exit potential (CXP) for each phenolic compound. Data acquisition was carried out with the Analyst 1.6.2 software (AB Sciex, USA). The phenol metabolites in the blood samples were identified by comparing their spectra and retention times (*t*_R_) with those of externally injected standards. Compounds for which standards were not available were tentatively identified using MRM transitions with the mass of the parent ion (M–H) and typical MS fragmentation patterns described in the literature. Some of the compounds were quantified using the calibration curves of their corresponding commercial standards. The other compounds were tentatively quantified using the calibration curves of standards with similar chemical structures. All calibration curves had correlation coefficients greater than 0.99 (*R* ^2^ > 0.99).

### 2.6 Statistics

Statistical analysis was performed using GraphPad Prism version 6.0 for Windows (GraphPad Software, Boston, MA, USA). The data were presented as means ± standard error of the mean (SEM) of independent measures performed in duplicate. A one‐way analysis of variance (ANOVA) was used to evaluate statistical differences (*p* < 0.05) in the iAUC means across experimental groups (WBB + sugar solution, water + sugar solution, and WBB + water), considering all volunteers and comparing responders and non‐responders. When significant differences were detected, a post hoc Tukey's test was applied to identify pairwise differences between groups. In addition, the linear relationship between blood glucose levels and blood phenolic metabolite concentrations during the postprandial period was evaluated using Pearson's correlation coefficient (*r*). Correlations were interpreted as weak (*r* < 0.4), moderate (0.4 and 0.8), or strong (*r* > 0.8).

## 3 Results

### 3.1 Sensory Evaluation and Selection of the Wine By‐Product‐Based Beverage (WBB)

Four formulations combining grape skins and seeds from grape‐pomace in different proportions were prepared for the selection of the WBB for the postprandial blood glycemia study. The selection criteria were based on the combination of the diverse and high content of (poly)phenols but also attractive for their organoleptic properties. Table 1 shows the (poly)phenol content in 200 mL of each WBB formulation, corresponding to the dose ingested through each intervention (acute intake) (Figure 1). A wide diversity of (poly)phenols were quantified by UHPLC‐MS/MS, the anthocyanins being the main fraction, mainly in Formulations 2 and 4, corresponding to the higher percentage of the skin fraction used as the main ingredient (Table 1). Regarding noncolored phenols, flavonols were the predominant subfamily, including quercetins and myricetins, and flavan‐3‐ols, including (epi)catechins and procyanidins (Table 1). The soluble fiber (pectin) content was related with a higher percentage of grape skin in the formulation, corresponding to Formulas 2 and 4, with a total of 10% and 8%, respectively.

Besides the (poly)phenol and the soluble fiber content, the selection of the WBB formulation was further based on their sensory evaluation. Data for each evaluated attribute (appearance, odor, taste, and texture, as well as overall quality) along with their respective sensory descriptors is presented in Table 2, offering insights into key differences and consumer perceptions across the four formulations. Appearance was primarily influenced by brightness with statistically significant differences (*p* < 0.05) observed only between Formulations 1 and 4, the later showing more a pronounced effect, similarly to the elevated perception of a sandy texture (Table 2). Regarding odor, the evaluated descriptors did not indicate significant differences between formulations. In general, low values were obtained, suggesting a nonspecific aroma characteristic of the four WBB formulations. The gustatory profile was strongly influenced by acidity, with significant differences between Formulations 2 (10% of grape skins) and 3 (4% of grape skins), corresponding to the products with the highest and lowest percentage of skins, respectively. However, trends in specific attributes were noted, leading to similar scores across the four WBB formulations.

**TABLE 2:** Formulations of wine byproducts‐based beverages (WBB) elaborated for sensorial analysis.

Formulation	1	2	3	4
Sensory descriptors^*^	Mean ± SD	Mean ± SD	Mean ± SD	Mean ± SD
Appearance				
Brightness	**4.80** ± **2.10^b^**	**6.20** ± **1.29^ab^**	**5.37** ± **1.56^ab^**	**7.04** ± **1.61^a^**
Pink‐violet	7.55 ± 1.32	7.78 ± 1.54	6.16 ± 2.00	7.81 ± 1.52
Homogeneity	5.99 ± 2.46	6.79 ± 2.50	7.44 ± 1.56	6.10 ± 1.98
Density	5.84 ± 2.46	6.65 ± 2.03	4.98 ± 2.74	6.88 ± 1.78
Sandy	7.51 ± 1.48	7.33 ± 1.58	6.15 ± 2.67	6.81 ± 1.72
Odor				
Pressed grape skin	4.46 ± 2.55	5.81 ± 2.29	4.65 ± 1.63	5.20 ± 2.58
Overall intensity	5.77 ± 2.49	5.67 ± 1.96	4.81 ± 2.53	6.64 ± 1.53
Overall quality	5.62 ± 2.46	5.88 ± 1.57	4.20 ± 2.50	6.35 ± 1.74
Grape stem	3.54 ± 2.75	3.67 ± 2.91	3.01 ± 2.09	3.07 ± 2.48
Herbaceous	3.95 ± 2.75	2.82 ± 2.73	2.80 ± 2.51	3.12 ± 2.56
Acidic	2.62 ± 2.51	2.91 ± 2.86	2.61 ± 2.73	2.69 ± 2.60
Red berries	3.67 ± 3.23	2.96 ± 2.66	2.85 ± 2.87	3.59 ± 3.38
Unripe fruit	3.54 ± 3.23	2.90 ± 2.79	2.89 ± 2.59	2.51 ± 2.59
Citric	1.85 ± 1.89	1.49 ± 1.23	2.15 ± 2.47	1.95 ± 1.38
Floral	2.67 ± 3.32	3.22 ± 2.78	3.06 ± 3.06	4.37 ± 3.11
Taste				
Overall quality	4.50 ± 2.59	4.90 ± 2.59	4.46 ± 1.70	4.91 ± 2.03
Sweet	2.28 ± 1.92	1.75 ± 1.78	1.84 ± 1.38	1.83 ± 2.19
Salt	1.23 ± 1.56	1.69 ± 2.51	2.17 ± 2.7	1.53 ± 2.39
Acid	**5.30** ± **3.33^ab^**	**6.63** ± **2.95^a^**	**3.45** ± **2.07^b^**	**6.18** ± **2.73^ab^**
Bitter	2.90 ± 3.24	3.31 ± 3.45	2.46 ± 3.14	3.20 ± 3.38
Mouth feel (texture)				
Astringence	3.60 ± 2.24	4.26 ± 2.72	3.07 ± 2.50	4.51 ± 2.70
Body	5.42 ± 2.48	6.73 ± 1.65	4.22 ± 2.50	5.86 ± 2.49
Persistency	4.88 ± 2.45	6.62 ± 1.56	3.98 ± 2.07	6.24 ± 1.78
Graininess	3.19 ± 2.65	3.78 ± 3.16	3.12 ± 2.58	4.83 ± 3.32
Sandy	**5.23** ± **2.76^a^**	**7.08** ± **2.03^ab^**	**4.87** ± **2.90^a^**	**12.9** ± **6.8^b^**
Global quality	*5.00* ± *1.90*	*5.04* ± *1.78*	*4.66* ± *1.77*	*5.38* ± *1.87*

*Note*: Mean hedonic valuation for consumer acceptance (*n* = 10) of the different sensory descriptors for the WBB formulations.

* Different letters in the same row indicated significant differences (*p* < 0.05).

*Note*: Number in bold reach statistical significance.

*Note*: Numer in italic represent the global quality of WBB considering all the studied attributes of WBB.

Based on the results of the sensorial analysis, except for perceptions of brightness and acidity, varying the by‐product supplementation within the 5%–10% range did not significantly alter the sensory attributes of the beverages. Thus, incorporating up to 10% of wine by‐products (Formulas 2 and 4) did not negatively impact the sensory attributes compared with the Formulas 1 and 3 with 5% of grape‐wine by‐products (Table 1). The brightness of the beverage (Table 2) could primarily be attributed to the (poly)phenol compounds retained in the grape skin after vinification, especially anthocyanins (Table 1). It appears that a higher grape skin content enhances the brightness of the WBB beverage, but this effect is noticeable only in the presence of grape seeds. This may indicate an interaction that potentiates brightness [12]. Apart from inducing brightness, phenolic compounds also affect the food taste, often contributing to bitterness and astringency [12]. In addition, a marked perception of acidity may be due to the low or no sugar content in the grape pomace after alcoholic fermentation during winemaking. Detailed information on intensity (*I*, %), frequency (*F*, %), and global media (GM, %) of each WBB is provided in Table S3.

Thus, the consumer acceptance data from the four WBB beverages tested guided our decision to select Formulation 4 (Table 1) for the pilot human study. The WBB selected contains 8% of grape skins and 2% of seeds. Thus, a 200 mL serving of this WBB provides an average of 187 mg of total (poly)phenols, particularly anthocyanins (132 mg), flavonols (24 mg), and flavan‐3‐ols (17.2 mg) and 664 mg of soluble fiber (pectin) (Table 1).

### 3.2 Glucose Overload Human Study: Postprandial Blood Glycemia

**FIGURE 2:**
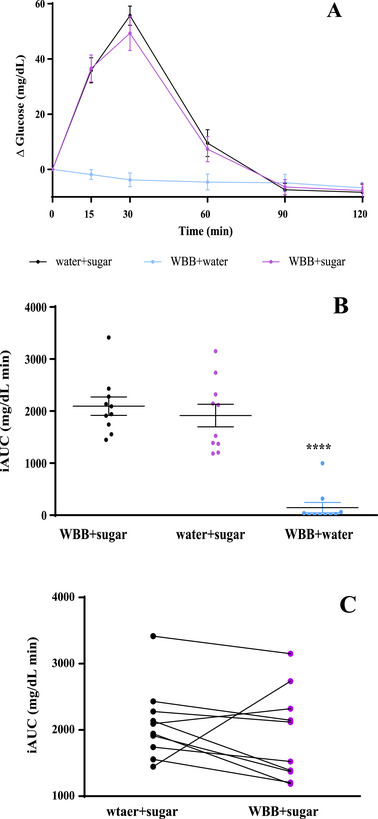
(A) Incremental blood glucose over 2‐h of postprandial period. Mean ± standard error of the mean (SEM), *n* = 10. (B) Incremental area under the curve (iAUC) for blood glucose. Mean ± SEM, *n* = 10. (C) Variation of the iAUC between water + sugar solution and wine by‐products‐based beverage (WBB) + sugar solution. ****p < 0.0001 versus WBB + sugar and water + sugar solution.

To evaluate the hypoglycemic potential of the WBB in the postprandial state, we analyzed both the time course of blood glucose levels and the iAUC for each acute intervention (Figure 1). Figure 2A shows the incremental blood glucose (mean ± SEM, *n* = 10) at various time points (0, 15, 30, 60, 90, and 120 min) during the postprandial period. As expected, no increase in blood glucose was observed when participants consumed the WBB + water (i) (Figure 1), indicating the negligible sugar content in the beverage as a result of alcoholic fermentation during the winemaking process, making this product suitable for prediabetic and diabetic individuals. When comparing the two glucose overload interventions (Figure 1ii and iii), a typical pattern of blood glucose increase previously described in healthy adults was observed, with levels peaking 30 min after sugar intake and then gradually returned to the baseline [13, 14]. In this context, in our study, the blood glucose level at 30 min tended to be lower when the sugar solution was consumed in combination with the WBB. However, this difference was not statistically significant.

The iAUC for blood glucose [15] calculated for each acute intervention is depicted in Figure 2B. This iAUC reflects the time‐averaged glucose concentrations accumulated over the 2‐h postprandial period. A lower iAUC was observed following the WBB + water intake compared to the water + sugar solution. However, this difference was not statistically significant. This was probably a consequence of the interindividual variability in blood glucose responses after each treatment, as illustrated in Figure 2C, which shows the individual variation in the iAUC between the water + sugar solution and WBB + sugar solution interventions (Figure 1). These findings led us to establish two groups: individuals who respond to the intake of the WBB beverage treatment (“responders”) and “non‐responders”, showing varying responses to the sugar overload.

### 3.3 Individual Blood Glucose Variability Over 2‐h of the Postprandial Period: Responders and Non‐Responders

**FIGURE 3:**
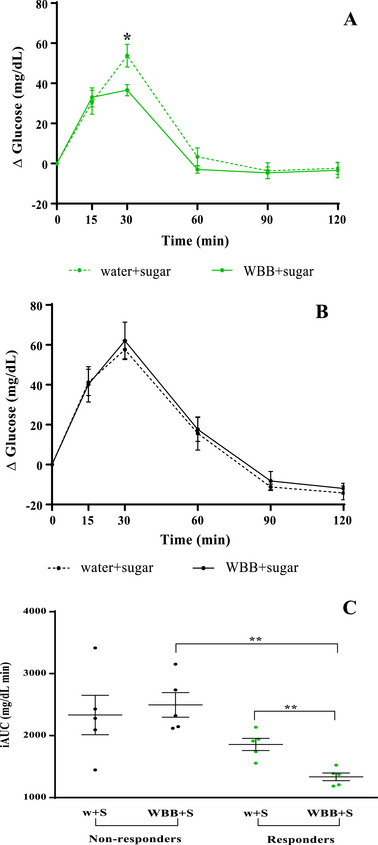
Time course of incremental blood glucose following sugar challenge in responders, *n* = 5 (A) and non‐responders, *n* = 5 (B); (C) incremental area under the curve (iAUC) in responders and non‐responders after the intake of water + sugar solution (w + S) and wine by‐products‐based beverage (WBB) + sugar solution (WBB + S). Data are expressed as mean ± standard error of the mean (SEM). **p<0.01.

Individual analysis (Figure 2C) revealed that some volunteers responded positively to the WBB treatment, while others showed no response and even, in the case of one volunteer, the contrary effect was observed. Based on these observations, we identified two distinct groups: “responders” (*n* = 5), who experienced a reduction of more than 10% in their iAUC after the intake of the WWB combined with the sugar solution, and “non‐responders” (*n* = 5). After categorizing the volunteers into these two groups, we compared the blood glucose time course between “respondents” and “non‐responders” for both sugar interventions (water + sugar solution and WBB + sugar solution). As shown in Figure 3A, the responders exhibited a notable reduction in incremental glucose levels following the intake of the WBB + sugar solution, reaching statistical significance at 30 min when glucose levels peaked. In contrast, no significant differences in incremental blood glucose were observed at any time during the postprandial period in the non‐responders’ group (Figure 3B). In addition, in the responder's group, significant differences in the iAUC were observed when comparing treatments with the water + sugar solution with the sugar solution combined with the WBB. However, in the group of non‐responders, no statistically significant differences were observed (Figure 3C). This decrease appears to be associated with the WBB intake, as the iAUC values for the water + sugar solution treatments were similar for “responders” and “non‐responders”.

### 3.4 Blood Phenol Biological Metabolites During the Postprandial Period After the Sugar Overload

**FIGURE 4:**
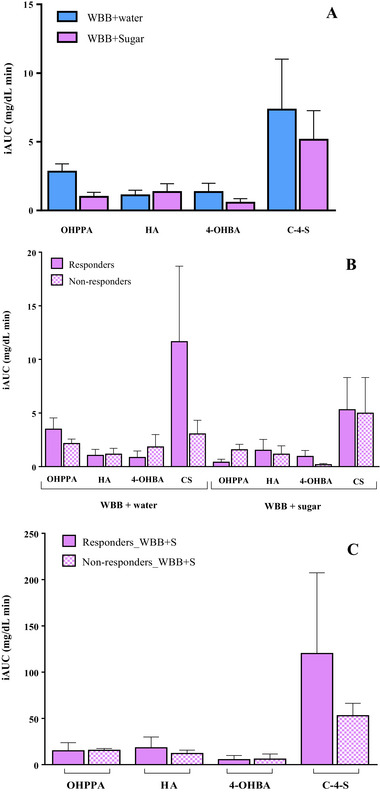
Incremental area under the curve (iAUC) for each of the four phenol metabolites detected in blood during the postprandial period (A) 0–120 min after the intake of WBB + water and WBB + sugar solution in all volunteers (*n* = 10), (B) 0–120 min after the intake of WBB + water and WBB + sugar solution in responders (*n* = 5) and non‐responders (*n* = 5), (C) 0–12 h after the intake of WBB + water and WBB + sugar solution in responders (*n* = 5) and non‐responders (*n* = 5). Data are expressed as mean ± SEM. 4‐OHBA, 4‐hydroxybenzoic acid; CS, catechol‐4‐sulfate; HA, hippuric acid; OHPPA, hydroxyphenyl propionic acid; SEM, standard error of the mean; WBB, wine by‐products‐based beverage.

Identifying (poly)phenol intake biomarkers in blood samples following sugar loading may offer valuable insights into the role of these compounds in the regulation of postprandial hyperglycemia. Based on this premise, we proposed to investigate if differences in the glucose response observed between “responders” and “non‐responders” could be related to differences in the bioavailability and/or metabolism of (poly)phenols from the WBB. For this, we analyzed blood samples collected on DBS cards at different time points (0, 15, 30, 60, 90, and 120 min and 4, 6, and 12 h) following interventions with the WBB + water and WBB + sugar solutions (Figure 1). The UPLC‐MS/MS analysis of the blood samples from both interventions revealed the presence of four main phenolic metabolites: hydroxyphenyl propionic acid (OHPPA), HA, 4‐hydroxybenzoic acid (4‐OHBA), and catechol‐4‐sulfate (CS) (Figure 4A). To normalize the data, the iAUC was calculated for each of the four phenol metabolites detected in blood during postprandial period. No statistically significant differences were observed in the iAUC of these phenol metabolites when the WBB was consumed with water or with the sugar solution, respectively, suggesting that the absorption and/or metabolism of phenolic compounds was not affected by the postprandial glycemia.

Following the identification of blood phenolic metabolites, we aimed to explore differences in their concentrations and behavior between the “responders” and “non‐responders”. As shown in Figure 4B, no significant differences in the blood concentration of OHPPA, HA, 4‐OHBA and CS were observed during the postprandial period (0–120 min) between the “responders” and “non‐responders”, after the intake of both the WBB + water and WBB + sugar solution treatments, respectively. To further investigate, the analysis was extended to blood phenolics in the later postprandial phase (4–12 h), corresponding to colonic microbial metabolism of the WBB (poly)phenols (Figure 4C). It should be noted that the iAUC of the HA and the C‐4‐S was higher in the responders, although no statistically significant differences were observed, probably related with the high interindividual variability.

## 4 Discussion

**FIGURE 5:**
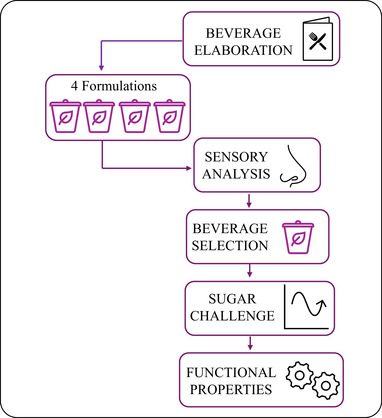
Flow chart of the beverage design and functional evaluation.

This study derives from the interest in the valorization of the winemaking by‐products characterized in a previous work that revealed their great potential as a source of phenolic compounds [5]. Considering the growing health concerns related to altered glucose metabolism, we assume this work as a proof‐of‐concept to evaluate the applicability of a WBB as a functional food for controlling postprandial hyperglycemia. Our aim was to explore the potential of this beverage for regulating blood glucose levels following an acute intake of a sugar solution in fasting conditions. Our research is aligned with the following primary objectives: promoting metabolic health through functional foods and advancing environmental sustainability by applying the principles of the circular bioeconomy. The present study was based on the hypothesis that consuming this beverage alongside a high‐sugar meal could help regulate postprandial glucose levels, potentially mitigating the long‐term damage associated with repeated hyperglycemic episodes [9]. For this, a comprehensive workflow was designed, which included product development, beverage formula selection based on the sensory analysis, and completed with a functional evaluation (Figure 5). To focus on wine by‐products, we used a simple beverage formulation that included only wine by‐products and a thickener to improve the stability and consistency of the beverage (Table 1). However, the wine by‐product ingredients could also be incorporated into more complex beverages to enhance their nutritional and functional value by increasing the concentration and diversity of the (poly)phenols and the soluble fiber (pectin) content. In general, technological refinements are needed to optimize the sensory quality of beverages enriched with food by‐products [2]. Nevertheless, the WBB developed in the present study was well‐accepted by the tasters. Consumers’ acceptance of wine by‐products is a key point in the successful market integration of functional foods elaborated from these by‐products. However, it is important to take into account that acceptance is influenced by multiple factors beyond organoleptic properties. One significant aspect is consumer awareness of the origin and benefits of wine by‐products consumption, which needs educational initiatives to communicate their advantages. Positive aspects include contribution to the bioeconomy by promoting sustainability through the consumption of eco‐friendly foods, as well as the intake of bioactive compounds, especially polyphenols and dietary fiber that provide additional health benefits.

In recent years, promising results have been observed regarding the efficacy of wine by‐products alone or in combination with other phenolic‐rich plant by‐products to control blood glucose levels [6]. Several mechanisms, sometimes acting simultaneously, have been proposed for postmeal blood glucose regulation by (poly)phenols. These include the inhibition of digestive enzyme activity, the reduction of glucose uptake by intestinal cells, the increase of glucose utilization by cells through the stimulation of insulin secretion and enhancement of insulin resistance, and the modulation of glucoregulatory intracellular signaling pathways [3, 4, 6, 7, 16, 17].

In the present study, after comparing the blood glucose levels following the intake of the WBB + sugar and water + sugar solutions, we observed that some volunteers responded positively, showing a decrease in the iAUC (0–120 min), while others showed no change or unexpected increases in glucose levels (Figure 2C). We hypothesized that interindividual variation in the WBB response could be associated to metabolic differences during the postprandial period, possibly affected by individual differences in the phenolic compound absorption and/or metabolism. To explore this, we studied the presence of the phenolic metabolites in blood samples from the volunteers at the same time‐points of glucose measurement (0–120 min) and also 4, 6, and 12 h postingestion in order to determine the microbial colonic metabolites. Identifying specific phenolic metabolites in blood could explain the interindividual differences in the reduction of the glycemic response after the intake of the sugar solution combined with the WBB.

Based on the results of this study (Figure 4), we hypothesize that the phenolic metabolites detected in blood using DBS cards are not directly involved in glucoregulatory mechanisms in acute postprandial glycemia. This is supported by the lack of a clear association between their blood concentration during the postprandial period and the observed reduction in blood glucose levels in responder volunteers (data not shown). However, we hypothesized that this lack of the relationship could be due to the analytical method that combines DBS blood card sampling with chromatographic analysis, which could not be sensitive enough to detect minor phenol metabolites that could be responsible for the hypoglycemic effects of the WBB.

Several studies have evaluated the lowering postprandial glucose effect of phenolic‐rich products [8, 18], but among them, a small proportion have also evaluated the circulating phenolic metabolites postingestion in humans. A study by Prpa et al. [4] determined the serum concentration of phenol metabolites after the intake of apple juice enriched with different doses of apple extract. At 120‐ and 240‐min postingestion, a dose–response effect on the concentration of five phenol metabolites was observed, but no differences in the iAUC were probably related with the high interindividual variability. This may indicate that after acute intake interventions, the phenolic compounds present in the circulation may not be involved in the regulation of postprandial glucose levels. Many studies have attributed the hypoglycemic effect of phenolic compounds to their capacity to interfere with the hydrolytic activity of digestive enzymes, reducing the release of glucose to the intestinal lumen and thus decreasing its absorption [8].

Nevertheless, the regular and sustained intake of phenolic‐rich products may contribute to maintaining a steady concentration of circulating phenolic species due to hepatic Phases I and II metabolism with an additional contribution from enterohepatic recirculation [19]. In the present study, we aimed to explore the potential role of microbial metabolites from wine by‐products in the bloodstream to assess their possible contribution to long‐term hypoglycemic effects. Our findings indicated an increase in the total concentration of phenolic microbial metabolites over the 4–12 h postingestion compared to the postprandial period (0–2 h). However, no statistically significant differences were observed between the “responder” and “non‐responder” volunteers. These results, nevertheless, do not invalidate the potential regulatory effects of circulating phenolic metabolites on postprandial glycemia, particularly with a regular and sustained consumption of phenolic‐rich products [20].

The hypoglycemic effect of the WBB beverage observed in the present study could also be related to the presence of pectin (soluble dietary fiber). The short‐term hypoglycemic effect of dietary fiber is due to its physicochemical properties, which can hinder glucose absorption and/or carbohydrate digestion by increasing the viscosity of the digesta [21]. Moreover, dietary fiber also promotes glycemic control over the long term by favoring the development of beneficial intestinal bacteria and enhancing the production of the bioactive microbial metabolites [22]. This glucoregulatory effect typically requires regular and sustained fiber consumption to maximize its hypoglycemic effects. For instance, pectin, a type of soluble fiber found in wine by‐products, may be involved in the modulation of glucose absorption during digestion [1, 23]. However, there is limited information regarding the possible role of pectin in managing postprandial glycemia, highlighting the need for further studies and a better understanding of its hypoglycemic properties.

Our results support the theory that the WBB may have acute hypoglycemic effects in certain individuals (“responders”), likely due to the interaction of phenolic compounds and fiber in the intestinal lumen during digestion. The magnitude of the glucose‐lowering properties seems to be influenced by factors within the digestive process, rather than by the bioavailable (poly)phenols. Although our findings are derived from an acute intake intervention study, we cannot overlook the potential benefits of regular consumption of phenolic‐rich products, which may offer long‐term advantages through the continuous release of phenol metabolites, including those provided by the gut microbiota. These microbial metabolites may contribute to sustained improvements in glucose regulation over time.

This proof‐of‐concept pilot study included 10 healthy volunteers, with 5 classified as responders, aiming to assess feasibility and refine methodologies for future large trials. The observed variability in responses highlights the need for personalized nutrition approaches and justifies the small‐scale, resource‐efficient design before larger studies. A deeper understanding of the factors that drive the variation between individuals in the metabolic response to dietary modifications may guide more effective and precise dietary recommendations for individuals in clinical practice. This highlights the importance of precision nutrition in understanding these differential responses.

## 5 Conclusions

The results of this study suggest that wine by‐products can be used effectively as ingredients in the formulation of functional beverages. These ingredients can be incorporated into both solid and liquid formulations to enhance the concentration of bioactive compounds. In the context of sugar overload in healthy adults, our findings indicate that the intake of a WBB beverage may help reduce postprandial hyperglycemia in some individuals who can be considered as “responders”. Additionally, this study highlights the potential of wine by‐products in managing glucose iAUC and variations in the postprandial maximum glucose level in healthy subjects. This emphasizes the role of natural products in supporting glucose management and promoting metabolic health. Moreover, wine by‐products can be combined with other fruit or vegetables to create a range of beverages, further enhancing their bioactive profile while maintaining sensory acceptance and providing added health and nutritional benefits as sources of a wide diversity of (poly)phenols and soluble fiber (pectin). The results of this proof‐of‐concept study demonstrate a significant potential for the valorization of by‐products from the winemaking process, promoting their reutilization and valorization within a circular bioeconomy model.

## Conflicts of Interest

The authors declare no conflicts of interest.

## Supporting information



Supporting information

Supporting information

Supporting information

Supporting information

## Data Availability

The data supporting this article have been included as part of the Supporting Information (Figure S1 and Tables S1–S3).
