# Supplementary figures and images for: Development of a Wine By‐Product‐Based Beverage and Study of Its Potential to Postprandial Glycemia Regulation in Healthy Individuals: A Proof of Concept Study
Source: Mol Nutr Food Res. 2025 May 27;69(14):e70128. doi: 10.1002/mnfr.70128 (PMC12280843; doi:10.1002/mnfr.70128)

## Slide 1
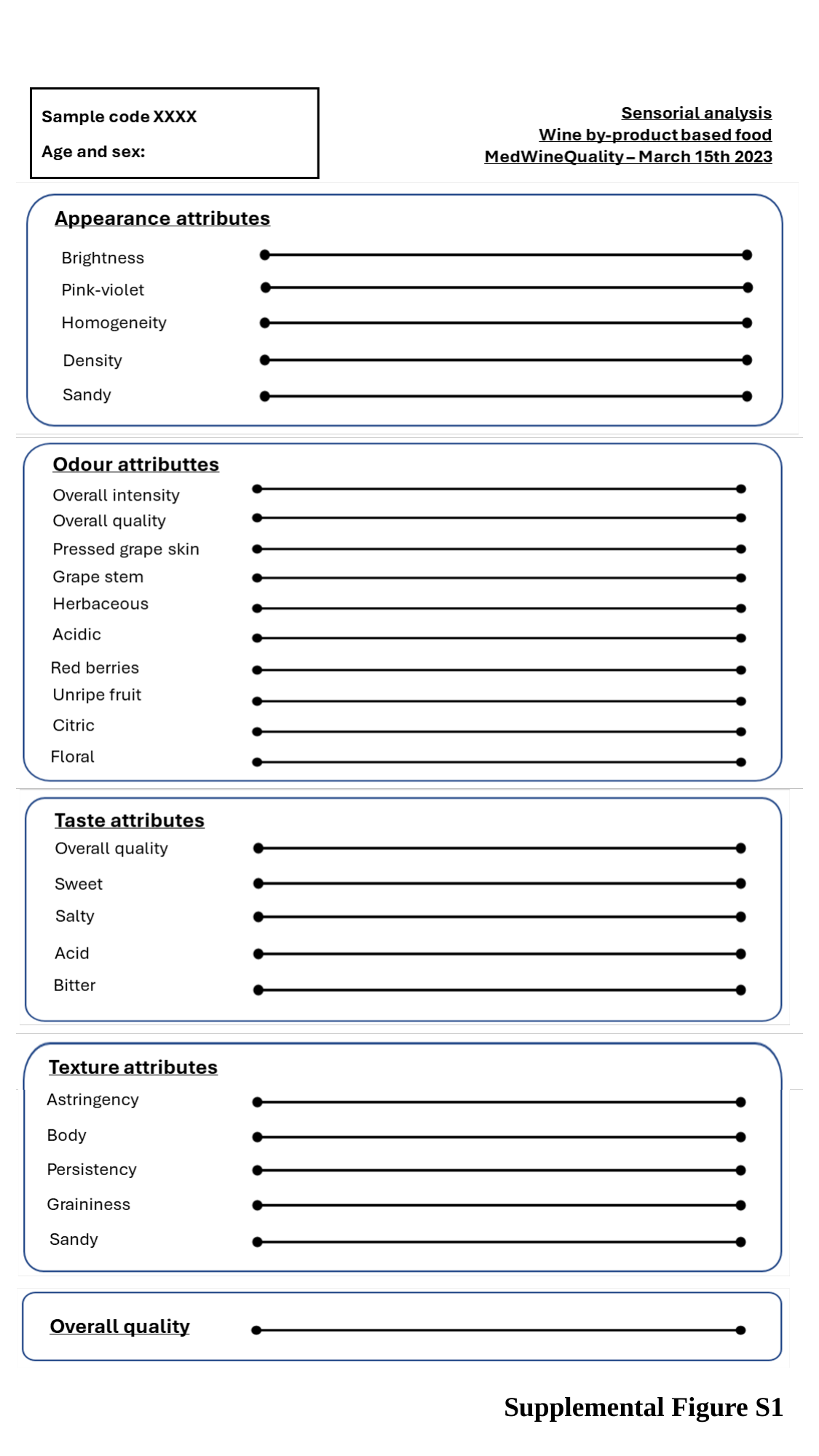

Supplemental Figure S1

Supplement: Supplementary file 1 — Supporting information [file MNFR-69-e70128-s002.pptx]
