# Supplementary material for: Development of a Wine By‐Product‐Based Beverage and Study of Its Potential to Postprandial Glycemia Regulation in Healthy Individuals: A Proof of Concept Study
Source: Mol Nutr Food Res. 2025 May 27;69(14):e70128. doi: 10.1002/mnfr.70128 (PMC12280843; doi:10.1002/mnfr.70128)
Supplement: Supplementary file 2 — Supporting information [file MNFR-69-e70128-s003.docx]

**Supplemental Table S1**. Quali-quantitative phenolic profile of byproducts from red Tempranillo and Graciano grapes used for the beverage elaboration. Data is expressed as mean ± SD (n=2).

| Phenolic compound | Skin | | | | | | Seed | | |
| --- | --- | --- | --- | --- | --- | --- | --- | --- | --- |
| (mg/kg dry material) | Graciano | | | Tempranillo | | | Tempranillo | | |
| Malvidins | 6425 | ± | 469 | 2704 | ± | 7 | 191 | ± | 24 |
| Petunidins | 1444 | ± | 78 | 475 | ± | 0.2 | 26 | ± | 3.8 |
| Delphinidins | 2419 | ± | 85 | 695 | ± | 5 | 34 | ± | 4 |
| Peonidins | 1596 | ± | 118 | 145 | ± | 0.0 | 9 | ± | 1.46 |
| Cyanidins | 293 | ± | 16 | 51 | ± | 0.2 | 4 | ± | 0.4 |
| Pelargonidins | 2.9 | ± | 0.1 | 0 | ± | 0.0 | 0 | ± | 0.0 |
| Minor compounds | 47 | ± | 2 | 55 | ± | 0.7 | 3 | ± | 0.3 |
| *Total colored compounds (anthocyanidins)* | *12225* | *±* | *769* | *4124* | *±* | *11.4* | *268* | *±* | *34.7* |
| Hydroxycinnamic acids | 104 | ± | 9 | 248 | ± | 13 | 87 | ± | 9 |
| Hydroxybenzoic acids | 179 | ± | 12 | 247 | ± | 2 | 716 | ± | 117 |
| *Total Phenolic acids* | *282* | *±* | *21* | *495* | *±* | *11* | *802* | *±* | *127* |
| *Total Phenyl alcohols* | *99* | *±* | *25* | *123* | *±* | *10* | *78* | *±* | *9* |
| *Total Flavanones* | *4.5* | *±* | *0.2* | *3.60* | *±* | *0.07* | *1* | *±* | *0.2* |
| Myricetins | 431 | ± | 32 | 156 | ± | 4 | 11 | ± | 1 |
| Quercetins | 1152 | ± | 92 | 903 | ± | 6 | 77 | ± | 14 |
| Minor compounds | 142 | ± | 9 | 130 | ± | 2 | 8 | ± | 1 |
| *Total Flavonols* | *1726* | *±* | *133* | *1189* | *±* | *13* | *95* | *±* | *16* |
| Catechins | 175 | ± | 8 | 193 | ± | 2 | 1041 | ± | 151 |
| Procyanidins | 255 | ± | 17 | 335 | ± | 9 | 1224 | ± | 193 |
| *Total Flavon-3-ols* | *430* | *±* | *9* | *528* | *±* | *7* | *2265* | *±* | *344* |
| *Total Stilbenes* | *83* | *±* | *2* | *11.2* | *±* | *0.1* | *3.5* | *±* | *0.5* |
| *Total Lignans* | *23.9* | *±* | *0.9* | *63* | *±* | *3* | *178* | *±* | *24* |
| *Total non-colored phenols* | *2649* | *±* | *191* | *2414* | *±* | *5* | *3423* | *±* | *503* |
| *TOTAL PHENOLIC COMPOUNDS* | *14874* | *±* | *960* | *6538* | *±* | *7* | *3691* | *±* | *538* |
