# Supplementary material for: Development of a Wine By‐Product‐Based Beverage and Study of Its Potential to Postprandial Glycemia Regulation in Healthy Individuals: A Proof of Concept Study
Source: Mol Nutr Food Res. 2025 May 27;69(14):e70128. doi: 10.1002/mnfr.70128 (PMC12280843; doi:10.1002/mnfr.70128)
Supplement: Supplementary file 3 — Supporting information [file MNFR-69-e70128-s004.docx]

**Table S2.** Optimized MRM conditions for liquid chromatography analysis (HPLC-MS/MS) of the (poly)phenolic compounds determined in grape skins and seeds, and their biological metabolites determined in blood samples.

| **Compound** | **RT (min)** | **MRM transitions for quantification** | | | | | |  | **MRM transitions for identification** | | | | | | **Standard used to quantify** |
| --- | --- | --- | --- | --- | --- | --- | --- | --- | --- | --- | --- | --- | --- | --- | --- |
|  |  | **Q1 / Q3** | **DP** | **EP** | **CEP** | **CE** | **CXP** |  | **Q1 / Q3** | **DP** | **EP** | **CEP** | **CE** | **CXP** |  |
| **Anthocyanins^(1)^** | | | | | | | | | | | | | | | |
| Malvidin-3-glucoside | 7.30 | 493.1 / 331.2 | 56 | 10 | 22 | 29 | 6 |  | 493.1 / 315.2 | 56 | 10 | 22 | 57 | 6 | Malvidin-3-glucoside |
| Malvidin-3-(6"-acetyl)-glucoside | 9.90 | 535.0 / 331.1 | 60 | 8 | 25 | 35 | 14 |  | 535.0 / 315.2 | 60 | 8 | 25 | 50 | 6 | Malvidin-3-glucoside |
| *cis-*Malvidin-3-(6"-coumaroyl)- glucoside | 10.7 | 639.0 / 331.1 | 80 | 8 | 29 | 50 | 14 |  | 639.0 / 315.2 | 80 | 8 | 29 | 70 | 6 | Malvidin-3-glucoside |
| *trans-*Malvidin-3-(6"-coumaroyl)-glucoside | 11.2 | 639.0 / 331.2 | 80 | 8 | 29 | 50 | 14 |  | 639.0 / 315.2 | 80 | 8 | 29 | 70 | 6 | Malvidin-3-glucoside |
| Malvidin-3,5-diglucoside | 6.80 | 655.0 / 331.1 | 80 | 8 | 29 | 35 | 6 |  | 655.0 / 493.0 | 80 | 8 | 29 | 20 | 14 | Malvidin-3-glucoside |
| Malvidin-3-(6"-caffeoyl)-glucoside | 10.3 | 655.0 / 331.0 | 60 | 8 | 29 | 35 | 14 |  | 655.0 / 315.0 | 60 | 8 | 29 | 35 | 14 | Malvidin-3-glucoside |
| Malvidin-3-arabinoside | 7.60 | 463.0 / 331.1 | 36 | 8 | 23 | 35 | 14 |  | 463.0 / 315.2 | 36 | 8 | 23 | 57 | 6 | Malvidin-3-glucoside |
| Malvidin-3-glucoside-catechin | 4.70 | 781.0 / 619.0 | 50 | 10 | 33 | 30 | 6 |  | 781.0 / 603.0 | 50 | 10 | 33 | 50 | 6 | Malvidin-3-glucoside |
| Malvidin-3-glucoside-epicatechin | 6.30 | 781.0 / 619.0 | 50 | 10 | 33 | 30 | 6 |  | 781.0 / 603.0 | 50 | 10 | 33 | 50 | 6 | Malvidin-3-glucoside |
| Malvidin-3-glucoside-ethyl-catechin/epicatechin | 9.60 | 809.0 / 357.0 | 50 | 10 | 34 | 50 | 6 |  | 809.0 / 341.0 | 50 | 10 | 34 | 50 | 6 | Malvidin-3-glucoside |
| Malvidin-3,7-diglucoside | 5.80 | 655.0 / 331.0 | 80 | 8 | 29 | 35 | 6 |  | 655.0 / 493.0 | 80 | 8 | 29 | 20 | 6 | Malvidin-3-glucoside |
| Malvidin-3-glucoside-gallocatechin | 5.50 | 797.0 / 635.0 | 50 | 8 | 34 | 30 | 6 |  | 797.0 / 619.0 | 50 | 8 | 34 | 50 | 6 | Malvidin-3-glucoside |
| Malvidin-3-glucoside-ethyl-gallocatechin/epigallocatechin | 11.2 | 825.0 / 357.0 | 40 | 10 | 34 | 50 | 6 |  | 825.0 / 663.0 | 40 | 10 | 34 | 30 | 6 | Malvidin-3-glucoside |
| Malvidin-3-glucoside-4-vinylphenol | 11.5 | 609.0 / 447.0 | 50 | 8 | 28 | 40 | 6 |  | 609.0 / 431.0 | 50 | 8 | 28 | 60 | 6 | Malvidin-3-glucoside |
| Malvidin-3-glucoside-4-vinylguaiacol | 11.7 | 639.0 / 477.0 | 50 | 8 | 29 | 40 | 6 |  | 639.0 / 461.0 | 50 | 8 | 29 | 60 | 6 | Malvidin-3-glucoside |
| Malvidin-3-(6''-acetyl)-glucoside-4-ethyl-catechin/epicatechin | 10.5 | 851.0 / 357.0 | 50 | 8 | 35 | 40 | 6 |  | 851.0 / 341.0 | 50 | 8 | 35 | 60 | 6 | Malvidin-3-glucoside |
| Malvidin-3-(6''-acetyl)-glucoside-4-vinylcatechol | 11.5 | 667.0 / 463.0 | 50 | 8 | 30 | 40 | 6 |  | 667.0 / 447.0 | 50 | 8 | 30 | 60 | 6 | Malvidin-3-glucoside |
| Malvidin-3-(6''-acetyl)-glucoside-4-vinylphenol | 12.0 | 651.0 / 447.0 | 50 | 8 | 29 | 40 | 6 |  | 651.0 / 431.0 | 50 | 8 | 29 | 60 | 6 | Malvidin-3-glucoside |
| Malvidin-3-(6''-acetyl)-glucoside-4-vinylguaiacol | 11.9 | 681.0 / 477.0 | 50 | 8 | 30 | 40 | 6 |  | 681.0 / 461.0 | 50 | 8 | 30 | 60 | 6 | Malvidin-3-glucoside |
| Malvidin-3-(6''-coumaroyl)-glucoside-gallocatechin/epigallocatechin | 10.2 | 943.0 / 635.0 | 50 | 8 | 38 | 40 | 6 |  | 943.0 / 619.0 | 50 | 8 | 38 | 60 | 6 | Malvidin-3-glucoside |
| Malvidin-3-(6''-coumaroyl)-glucoside-catechin/epicatechin | 8.50 | 927.0 / 619.0 | 50 | 8 | 38 | 60 | 6 |  | 927.0 / 603.0 | 50 | 8 | 38 | 60 | 6 | Malvidin-3-glucoside |
| Malvidin-3-(6''-coumaroyl)-glucoside-ethyl-catechin/epicatechin | 10.2 | 955.0 / 357.0 | 50 | 8 | 39 | 40 | 6 |  | 955.0 / 341.0 | 50 | 8 | 39 | 60 | 6 | Malvidin-3-glucoside |
| Malvidin-3-(6''-coumaroyl)-glucoside-4-vinylcatechol | 12.0 | 771.0 / 463.0 | 50 | 8 | 33 | 40 | 6 |  | 771.0 / 447.0 | 50 | 8 | 33 | 60 | 6 | Malvidin-3-glucoside |
| Malvidin-3-(6''-coumaroyl)-glucoside-4-vinylphenol | 12.7 | 755.0 / 447.0 | 50 | 8 | 32 | 40 | 6 |  | 755.0 / 431.0 | 50 | 8 | 32 | 60 | 6 | Malvidin-3-glucoside |
| Malvidin-3-(6''-coumaroyl)-glucoside-4-vinylguaiacol | 12.9 | 785.0 / 477.0 | 50 | 8 | 33 | 40 | 6 |  | 785.0 / 461.0 | 50 | 8 | 33 | 60 | 6 | Malvidin-3-glucoside |
| Malvidin-3-(6''-acetyl-carbonyl-pyranoside)-glucoside | 8.70 | 603.0 / 399.0 | 50 | 8 | 28 | 40 | 6 |  | 603.0 / 383.0 | 50 | 8 | 28 | 60 | 6 | Malvidin-3-glucoside |
| Malvidin-3-(6''-coumaroyl-carbonyl-pyranoside)-glucoside | 10.1 | 707.0 / 399.0 | 50 | 8 | 31 | 40 | 6 |  | 707.0 / 383.0 | 50 | 8 | 31 | 60 | 6 | Malvidin-3-glucoside |
| Malvidin-3-(6''-acetyl-pyranoside)-glucoside | 9.30 | 559.0 / 355.0 | 50 | 8 | 26 | 40 | 6 |  | 559.0 / 339.0 | 50 | 8 | 26 | 60 | 6 | Malvidin-3-glucoside |
| Malvidin-3-(6''-coumaroyl-pyranoside)-glucoside | 10.6 | 663.0 / 355.0 | 50 | 8 | 29 | 40 | 6 |  | 663.0 / 339.0 | 50 | 8 | 29 | 60 | 6 | Malvidin-3-glucoside |
| Malvidin-3-glucoside-catechin | 5.50 | 797.0 / 635.0 | 50 | 8 | 34 | 40 | 6 |  | 797.0 / 619.0 | 50 | 8 | 34 | 50 | 6 | Malvidin-3-glucoside |
| Malvidin-3-glucuronide | 7.40 | 507.0 / 331.0 | 56 | 10 | 25 | 29 | 6 |  | 507.0 / 315.0 | 56 | 10 | 25 | 60 | 6 | Malvidin-3-glucoside |
| Malvidin-3-glucoside-glucuronide | 4.40 | 669.0 / 331.0 | 60 | 10 | 30 | 40 | 6 |  | 669.0 / 315.0 | 60 | 10 | 30 | 70 | 6 | Malvidin-3-glucoside |
| Malvidin-diglucuronide | 7.45 | 683.0 / 331.0 | 60 | 10 | 30 | 40 | 6 |  | 683.0 / 315.0 | 60 | 10 | 30 | 80 | 6 | Malvidin-3-glucoside |
| Malvidin-3-glucuronide sulphate | 8.70 | 587.0 / 331.0 | 60 | 10 | 27 | 40 | 6 |  | 587.0 / 315.0 | 60 | 10 | 27 | 90 | 6 | Malvidin-3-glucoside |
| Petunidin-3-glucoside | 6.15 | 479.1 / 317.0 | 51 | 9 | 22 | 29 | 6 |  | 479.1 / 302.1 | 51 | 9 | 22 | 47 | 6 | Petunidin-3-glucoside |
| Petunidin-3-(6"-acetyl)-glucoside | 9.30 | 521.0 / 317.0 | 60 | 8 | 25 | 50 | 14 |  | 521.0 / 302.0 | 60 | 8 | 25 | 70 | 6 | Petunidin-3-glucoside |
| *cis-*Petunidin-3-(6"-coumaroyl)-glucoside | 10.1 | 625.0 / 317.0 | 80 | 8 | 28 | 35 | 14 |  | 625.0 / 302.0 | 80 | 8 | 28 | 50 | 6 | Petunidin-3-glucoside |
| *trans-*Petunidin-3-(6"-coumaroyl)- glucoside | 10.3 | 625.0 / 317.0 | 80 | 8 | 28 | 35 | 14 |  | 625.0 / 302.0 | 80 | 8 | 28 | 50 | 6 | Petunidin-3-glucoside |
| Petunidin-3,5-diglucoside | 5.70 | 641.0 / 317.0 | 80 | 8 | 29 | 50 | 6 |  | 641.0 / 479.0 | 80 | 8 | 29 | 20 | 14 | Petunidin-3-glucoside |
| Petunidin-3-arabinoside | 6.30 | 449.0 / 317.0 | 36 | 8 | 23 | 35 | 14 |  | 449.0 / 302.0 | 36 | 8 | 23 | 50 | 6 | Petunidin-3-glucoside |
| Petunidin-3-glucuronide | 6.40 | 493.0 / 317.0 | 56 | 10 | 24 | 29 | 6 |  | 493.0 / 302.0 | 56 | 10 | 24 | 60 | 6 | Petunidin-3-glucoside |
| Petunidin-3-glucoside-glucuronide | 3.70 | 655.0 / 317.0 | 56 | 10 | 29 | 40 | 6 |  | 655.0 / 302.0 | 56 | 10 | 29 | 70 | 6 | Petunidin-3-glucoside |
| Delphinidin-3-glucoside | 4.45 | 465.0 / 303.0 | 50 | 7 | 23 | 30 | 4 |  | 465.0 / 229.0 | 50 | 8 | 24 | 70 | 4 | Delphinidin-3-glucoside |
| Delphinidin-3-(6"-acetyl)-glucoside | 7.90 | 507.0 / 303.0 | 60 | 8 | 24 | 50 | 14 |  | 507.0 / 229.0 | 60 | 8 | 24 | 70 | 4 | Delphinidin-3-glucoside |
| *cis-*Delphinidin-3-(6"-coumaroyl)- glucoside | 9.40 | 611.0 / 303.0 | 80 | 8 | 28 | 50 | 14 |  | 611.0 / 229.0 | 80 | 8 | 28 | 70 | 4 | Delphinidin-3-glucoside |
| *trans-*Delphinidin-3-(6"-coumaroyl)- glucoside | 9.80 | 611.0 / 303.0 | 80 | 8 | 28 | 50 | 14 |  | 611.0 / 229.0 | 80 | 8 | 28 | 70 | 4 | Delphinidin-3-glucoside |
| Delphinidin-3,5-diglucoside | 3.40 | 627.0 / 303.0 | 80 | 8 | 28 | 50 | 6 |  | 627.0 / 465.0 | 80 | 8 | 28 | 20 | 14 | Delphinidin-3-glucoside |
| Delphinidin-3-arabinoside | 6.30 | 435.0 / 303.0 | 35 | 7 | 22 | 30 | 4 |  | 435.0 / 229.0 | 36 | 8 | 22 | 70 | 4 | Delphinidin-3-glucoside |
| Delphinidin-3-glucuronide | 5.10 | 493.0 / 317.0 | 50 | 8 | 24 | 30 | 4 |  | 493.0 / 302.0 | 50 | 8 | 24 | 70 | 4 | Delphinidin-3-glucoside |
| Delphinidin-3-glucoside-glucuronide | 3.30 | 655.0 / 317.0 | 50 | 8 | 29 | 30 | 4 |  | 655.0 / 302.0 | 50 | 8 | 29 | 70 | 4 | Delphinidin-3-glucoside |
| Peonidin-3-glucoside | 7.00 | 463.1 / 301.2 | 56 | 10 | 22 | 29 | 6 |  | 463.1 / 286.1 | 56 | 10 | 22 | 47 | 4 | Peonidin-3-glucoside |
| Peonidin-3-(6"-acetyl)-glucoside | 9.80 | 505.0 / 301.0 | 60 | 8 | 24 | 35 | 14 |  | 505.0 / 286.0 | 60 | 8 | 24 | 50 | 6 | Peonidin-3-glucoside |
| *cis-*Peonidin-3-(6"-coumaroyl)- glucoside | 10.6 | 609.0 / 301.0 | 80 | 8 | 28 | 50 | 14 |  | 609.0 / 286.0 | 80 | 8 | 28 | 70 | 6 | Peonidin-3-glucoside |
| *trans-*Peonidin-3-(6"-coumaroyl)- glucoside | 10.9 | 609.0 / 301.0 | 80 | 8 | 28 | 50 | 14 |  | 609.0 / 286.0 | 80 | 8 | 28 | 70 | 6 | Peonidin-3-glucoside |
| Peonidin-3,5-diglucoside | 6.50 | 625.0 / 301.0 | 80 | 8 | 28 | 35 | 6 |  | 625.0 / 463.0 | 80 | 8 | 28 | 20 | 14 | Peonidin-3-glucoside |
| Peonidin-3-arabinoside | 7.05 | 433.0 / 301.0 | 36 | 8 | 22 | 35 | 6 |  | 433.0 / 286.0 | 80 | 8 | 22 | 50 | 6 | Peonidin-3-glucoside |
| Peonidin-3-glucuronide | 7.10 | 477.0 / 301.0 | 56 | 10 | 24 | 29 | 6 |  | 477.0 / 285.9 | 56 | 10 | 24 | 70 | 6 | Peonidin-3-glucoside |
| Peonidin-3-glucoside-glucuronide | 4.50 | 639.0 / 301.0 | 56 | 10 | 29 | 40 | 6 |  | 639.0 / 285.9 | 56 | 10 | 29 | 70 | 6 | Peonidin-3-glucoside |
| Peonidin-diglucuronide | 7.60 | 653.0 / 301.0 | 60 | 10 | 29 | 40 | 6 |  | 653.0 / 286.0 | 60 | 10 | 29 | 57 | 6 | Peonidin-3-glucoside |
| Methyl-peonidin-3-glucuronide-sulphate | 9.00 | 557.0 / 301.0 | 60 | 10 | 26 | 50 | 6 |  | 557.0 / 286.0 | 60 | 10 | 26 | 70 | 6 | Peonidin-3-glucoside |
| Cyanidin-3-glucoside | 5.70 | 449.1 / 287.1 | 51 | 9 | 20 | 29 | 4 |  | 449.1 / 137.1 | 51 | 9 | 20 | 73 | 4 | Cyanidin-3-glucoside |
| Cyanidin-(6"-acetyl)-glucoside | 8.90 | 491.0 / 287.0 | 80 | 8 | 24 | 50 | 4 |  | 491.0 / 213.0 | 80 | 8 | 24 | 50 | 4 | Cyanidin-3-glucoside |
| *cis-*Cyanidin-3-(6"-coumaroyl)- glucoside | 9.95 | 595.0 / 287.0 | 80 | 8 | 27 | 50 | 3 |  | 595.0 / 213.0 | 80 | 8 | 27 | 50 | 4 | Cyanidin-3-glucoside |
| *trans-*Cyanidin-3-(6"-coumaroyl)-glucoside | 10.3 | 595.0 / 287.0 | 80 | 8 | 27 | 50 | 4 |  | 595.0 / 213.0 | 80 | 8 | 27 | 50 | 4 | Cyanidin-3-glucoside |
| Cyanidin-3,5-diglucoside | 3.70 | 611.0 / 287.0 | 80 | 8 | 28 | 50 | 4 |  | 611.0 / 449.0 | 80 | 8 | 28 | 50 | 4 | Cyanidin-3-glucoside |
| Cyanidin-3-arabinoside A | 7.10 | 419.0 / 287.0 | 35 | 7 | 22 | 30 | 4 |  | 419.0 / 213.0 | 80 | 8 | 22 | 50 | 4 | Cyanidin-3-glucoside |
| Cyanidin-3-arabinoside B | 9.90 | 419.0 / 287.0 | 35 | 7 | 22 | 30 | 4 |  | 419.0 / 213.0 | 80 | 8 | 22 | 50 | 4 | Cyanidin-3-glucoside |
| Cyanidin-3-glucuronide | 5.70 | 463.0 / 287.0 | 56 | 10 | 23 | 29 | 6 |  | 463.0 / 137.0 | 56 | 10 | 23 | 60 | 6 | Cyanidin-3-glucoside |
| Pelargonidin-3,6-arabinoside | 6.60 | 403.0 / 271.0 | 80 | 10 | 21 | 50 | 4 |  | 403.0 / 271.0 | 80 | 10 | 21 | 50 | 4 | Cyanidin-3-glucoside |
| Pelargonidin-3,6-diglucoside | 6.50 | 433.0 / 271.0 | 80 | 10 | 22 | 50 | 4 |  | 433.0 / 271.0 | 80 | 10 | 22 | 50 | 4 | Cyanidin-3-glucoside |
| Vitisin A | 7.90 | 561.0 / 399.0 | 40 | 7 | 26 | 30 | 6 |  | 561.0 / 383.0 | 40 | 7 | 26 | 30 | 6 | Malvidin-3-glucoside |
| Vitisin B | 8.50 | 517.0 / 355.0 | 35 | 7 | 25 | 30 | 6 |  | 517.0 / 339.0 | 35 | 7 | 25 | 30 | 6 | Malvidin-3-glucoside |
| **Phenolic acids ^(2)^** | | | | | | | | | | | | | | | |
| ***Hydroxycinnamic acids*** |  |  |  |  |  |  |  |  |  |  |  |  |  |  |  |
| Caffeic acid | 5.40 | 178.8 / 134.2 | -25 | -6 | -14 | -40 | -2 |  | 178.8 / 135.1 | -25 | -6 | -14 | -18 | -2 | Caffeic acid |
| Caffeic acid ethyl ester | 12.7 | 207.0 / 132.9 | -65 | -10 | -14 | -42 | -2 |  | 207.0 / 179.0 | -65 | -10 | -19 | -20 | -2 | Caffeic acid ethyl ester |
| Caffeic acid sulphate | 5.40 | 259.0 / 179.0 | -40 | -8 | -21 | -25 | -2 |  | 259.0 / 135.0 | -40 | -8 | -21 | -40 | -2 | Caffeic acid |
| Caftaric acid | 3.70 | 311.0 / 179.0 | -60 | -5 | -20 | -30 | -2 |  | 311.0 / 135.1 | -60 | -5 | -23 | -50 | -2 | Caftaric acid |
| Caffeic acid-hexose | 4.30 | 341.0 / 135.0 | -60 | -5 | -20 | -50 | -2 |  | 341.0 / 179.0 | -60 | -5 | -20 | -30 | -2 | Caffeic acid |
| Chlorogenic acid  (3-*O*-caffeoylquinic acid) | 5.10 | 353.0 / 191.0 | -40 | -9 | -24 | -26 | -2 |  | 353.0 / 179.0 | -40 | -9 | -24 | -60 | -2 | Chlorogenic acid |
| 4-*O*-caffeoylquinic acid | 5.60 | 353.0 / 173.0 | -40 | -9 | -25 | -26 | -2 |  | 353.0 / 179.0 | -40 | -9 | -25 | -60 | -2 | Chlorogenic acid |
| *trans*-Coumaric acid | 7.20 | 162.9 / 119.0 | -20 | -10 | -14 | -12 | -2 |  | 162.9 / 92.8 | -20 | -10 | -18 | -38 | -2 | *trans*-Coumaric acid |
| *cis*-Coumaric acid | 7.80 | 162.9 / 119.0 | -20 | -10 | -14 | -12 | -2 |  | 162.9 / 92.8 | -20 | -10 | -18 | -38 | -2 | *trans*-Coumaric acid |
| Coutaric acid | 4.70 | 295.0 / 163.0 | -20 | -5 | -23 | -30 | -2 |  | 295.0 / 119.0 | -20 | -5 | -23 | -50 | -2 | Coutaric acid |
| Coumaric acid-hexose | 5.20 | 325.0 / 163.0 | -30 | -5 | -20 | -30 | -2 |  | 325.0 / 119.0 | -30 | -5 | -20 | -50 | -2 | *trans*-Coumaric acid |
| Coumaric acid sulphate | 5.70 | 243.0 / 163.0 | -40 | -4 | -21 | -18 | 2 |  | 243.0 / 119.0 | -40 | -4 | -21 | -38 | 2 | Coumaric acid sulphate |
| Hydrocoumaric acid | 7.40 | 164.8 / 147.0 | -35 | -10 | -18 | -20 | -2 |  | 164.8 / 119.1 | -35 | -10 | -18 | -20 | -2 | *trans*-Coumaric acid |
| *trans*-Ferulic acid | 8.30 | 193.0 / 134.0 | -25 | -9 | -19 | -16 | -2 |  | 193.0 / 178.1 | -25 | -9 | -19 | -12 | -4 | *trans*-Ferulic acid |
| *cis*-Ferulic acid | 9.00 | 193.0 / 134.0 | -25 | -9 | -19 | -16 | -2 |  | 193.0 / 178.1 | -25 | -9 | -19 | -12 | -4 | *trans*-Ferulic acid |
| Ferulic acid ethyl ester | 14.1 | 220.9 / 206.1 | -150 | -10 | -24 | -16 | -3 |  | 220.9 / 133.1 | -150 | -10 | -24 | -28 | -2 | Ferulic acid ethyl ester |
| Ferulic acid glucuronide | 5.60 | 369.0 / 193.0 | -50 | -6 | -25 | -18 | -2 |  | 369.0 / 134.0 | -50 | -6 | -25 | -32 | -2 | Ferulic acid sulphate |
| Ferulic acid sulphate | 6.90 | 273.0 / 193.0 | -45 | -6 | -22 | -18 | -2 |  | 273.0 / 134.0 | -45 | -6 | -22 | -32 | -2 | Ferulic acid sulphate |
| Isoferulic acid glucuronide | 7.00 | 369.0 / 193.0 | -50 | -6 | -25 | -18 | -2 |  | 369.0 / 134.0 | -50 | -6 | -25 | -32 | -2 | Ferulic acid sulphate |
| Isoferulic acid sulphate | 7.70 | 273.0 / 193.0 | -45 | -6 | -22 | -18 | -2 |  | 273.0 / 134.0 | -45 | -6 | -22 | -32 | -2 | Ferulic acid sulphate |
| *trans*-Fertaric acid | 5.80 | 325.0 / 193.0 | -35 | -8 | -24 | -18 | -3 |  | 325.0 / 134.0 | -35 | -8 | -24 | -34 | -2 | *trans*-Fertaric acid |
| ***Hydroxybenzoic acids*** |  |  |  |  |  |  |  |  |  |  |  |  |  |  |  |
| Gallic acid | 2.20 | 168.9 / 124.9 | -40 | -4 | -18 | -16 | 0 |  | 168.9 / 79.0 | -40 | -4 | -18 | -34 | 0 | Gallic acid |
| Gallic acid-glucoside | 3.10 | 331.0 / 124.9 | -60 | -5 | -24 | -50 | -2 |  | 331.0 / 124.0 | -60 | -5 | -24 | -50 | -2 | Gallic acid |
| Gallic acid sulphate | 3.20 | 249.0 / 169.0 | -40 | -8 | -21 | -25 | -2 |  | 249.0 / 125.0 | -40 | -8 | -21 | -40 | -2 | Gallic acid |
| Methyl-gallate | 3.70 | 183.0 / 124.0 | -40 | -4 | -18 | -16 | -2 |  | 183.0 / 168.9 | -40 | -6 | -18 | -10 | -2 | Gallic acid |
| Ethyl-gallate | 7.50 | 197.0 / 124.0 | -40 | -4 | -12 | -16 | -2 |  | 197.0 / 168.9 | -40 | -4 | -19 | -16 | -2 | Gallic acid |
| Syringic acid | 6.10 | 196.9 / 123.0 | -45 | -7 | -19 | -24 | -2 |  | 196.9 / 181.9 | -45 | -7 | -19 | -14 | -4 | Syringic acid |
| Syringic acid sulphate | 5.20 | 277.0 / 197.0 | -40 | -8 | -22 | -25 | -2 |  | 277.0 / 182.0 | -40 | -8 | -22 | -40 | -2 | Syringic acid |
| Syringic acid glucuronide | 4.50 | 373.0 / 197.0 | -40 | -8 | -25 | -25 | -2 |  | 373.0 / 182.0 | -40 | -8 | -25 | -40 | -2 | Syringic acid |
| Protocatechuic acid  (3,4-Dihydroxybenzoic acid) | 3.20 | 153.0 / 107.6 | -35 | -10 | -17 | -38 | -1 |  | 153.0 / 109.0 | -35 | -10 | -17 | -20 | -2 | Protocatechuic acid |
| Protocatechuic ethyl ester | 10.3 | 180.9 / 108.1 | -55 | -8 | -17 | -32 | -2 |  | 180.9 / 152.9 | -55 | -8 | -17 | -32 | -2 | Protocatechuic ethyl ester |
| Protocatechuic acid glucuronide | 6.90 | 329.0 / 153.0 | -40 | -8 | -24 | -25 | -2 |  | 329.0 / 109.0 | -40 | -8 | -24 | -40 | -2 | Protocatechuic acid |
| Protocatechuic acid sulphate | 3.90 | 233.0 / 153.0 | -40 | -8 | -20 | -25 | -2 |  | 233.0 / 109.0 | -40 | -8 | -20 | -40 | -2 | Protocatechuic acid |
| 4-Hydroxybenzoic acid | 4.10 | 137.0 / 93.0 | -35 | -9 | -17 | -22 | 0 |  | 137.0 / 64.9 | -35 | -9 | -17 | -42 | 0 | 4-Hydroxybenzoic acid |
| 4-Hydroxybenzoic acid sulphate | 4.10 | 217.0 / 137.0 | -40 | -8 | -20 | -25 | -2 |  | 217.0 / 93.0 | -40 | -8 | -20 | -40 | -2 | 4-Hydroxybenzoic acid |
| Sinapic acid-hexose | 8.80 | 385.0 / 223.0 | -40 | -6 | -26 | -15 | -2 |  | 385.0 / 205.0 | -40 | -6 | -26 | -30 | -2 | *trans*-Ferulic acid |
| Vanillic acid | 5.30 | 167.0 / 152.1 | -50 | -7 | -18 | -12 | -4 |  | 167.0 / 108.0 | -50 | -7 | -18 | -24 | -2 | Vanillic acid |
| Vanillic acid-hexose | 5.30 | 329.0 / 167.0 | -60 | -7 | -24 | -30 | -4 |  | 329.0 / 123.0 | -60 | -7 | -24 | -50 | -4 | Vanillic acid |
| Benzoic acid | 9.00 | 121.0 / 77.0 | -35 | -9 | -16 | -22 | 0 |  | 121.0 / 64.9 | -35 | -9 | -16 | -42 | 0 | 4-Hydroxybenzoic acid |
| 3-Hydroxybenzoic acid | 5.40 | 137.0 / 93.0 | -35 | -9 | -17 | -22 | 0 |  | 137.0 / 64.9 | -35 | -9 | -17 | -42 | 0 | 4-Hydroxybenzoic acid |
| 3,5-Dihydroxybenzoic acid | 3.00 | 152.9 / 109.0 | -35 | -10 | -17 | -20 | -2 |  | 152.9 / 107.6 | -35 | -10 | -17 | -38 | -1 | Protocatechuic acid |
| x,x-Dihydroxybenzoic acid | 3.70 | 152.9 / 109.0 | -35 | -10 | -17 | -20 | -2 |  | 152.9 / 107.6 | -35 | -10 | -17 | -38 | -1 | Protocatechuic acid |
| x,y-Dihydroxybenzoic acid | 4.10 | 152.9 / 109.0 | -35 | -10 | -17 | -20 | -2 |  | 152.9 / 107.6 | -35 | -10 | -17 | -38 | -1 | Protocatechuic acid |
| x,z-Dihydroxybenzoic acid | 5.20 | 152.9 / 109.0 | -35 | -10 | -17 | -20 | -2 |  | 152.9 / 107.6 | -35 | -10 | -17 | -38 | -1 | Protocatechuic acid |
| Phenylacetic acid | 9.20 | 135.0 / 91.0 | -30 | -9 | -17 | -12 | -2 |  | 135.0 / 107.0 | -30 | -9 | -17 | -24 | -2 | 4-Hydroxyphenylacetic acid |
| 2-Hydroxyphenylacetic acid | 6.40 | 151.0 / 106.9 | -30 | -9 | -17 | -15 | -2 |  | 151.0 / 64.7 | -30 | -9 | -17 | -28 | -2 | 4-Hydroxyphenylacetic acid |
| 3-Hydroxyphenylacetic acid | 5.80 | 151.0 / 106.9 | -30 | -9 | -17 | -15 | -2 |  | 151.0 / 64.7 | -30 | -9 | -17 | -28 | -2 | 4-Hydroxyphenylacetic acid |
| 4-Hydroxyphenylacetic acid | 4.90 | 151.0 / 106.9 | -30 | -9 | -17 | -15 | -2 |  | 151.0 / 64.7 | -30 | -9 | -17 | -28 | -2 | 4-Hydroxyphenylacetic acid |
| 3,4-Dihydroxyphenylacetic acid | 3.70 | 167.0 / 123.0 | -30 | -10 | -18 | -15 | -4 |  | 167.0 / 95.0 | -30 | -10 | -18 | -26 | -2 | 3,4-Dihydroxyphenylacetic acid |
| Hippuric acid | 5.00 | 178.0 / 134.0 | -40 | -10 | -18 | -15 | -2 |  | 178.0 / 77.0 | -40 | -10 | -18 | -20 | 0 | Hippuric acid |
| Phenylpropionic acid | 11.8 | 149.0 / 105.0 | -45 | -7 | -17 | -13 | -2 |  | 149.0 / 77.0 | -45 | -7 | -17 | -32 | -2 | 3-Phenylpropionic acid |
| 3-(3'-Hydroxyphenyl)propionic acid | 7.70 | 164.8 / 121.2 | -65 | -10 | -18 | -14 | -4 |  | 164.8 / 105.8 | -65 | -10 | -18 | -32 | -2 | 3-(3'-Hydroxyphenyl)propionic acid |
| 3-(4'-Hydroxyphenyl)propionic acid | 7.30 | 164.8 / 119.1 | -65 | -10 | -18 | -37 | -2 |  | 164.8 / 121.2 | -65 | -10 | -18 | -14 | -4 | 3-(3'-Hydroxyphenyl)propionic acid |
| 3-(3',4'-Dihydroxyphenyl)propionic acid (Hidrocaffeic acid) | 5.00 | 181.0 / 59.0 | -55 | -9 | -18 | -16 | -2 |  | 181.0 / 137.0 | -55 | -9 | -18 | -14 | 0 | 3-(3',4'-Dihydroxyphenyl)propionic acid |
| 4,4-Bis-4-hydroxyphenylvaleric acid | 11.9 | 285.0 / 147.0 | -80 | -10 | -22 | -24 | -2 |  | 285.0 / 93.0 | -80 | -10 | -22 | -26 | -2 | 4,4-Bis-4-hydroxyphenylvaleric acid |
| 4-Hydroxy-5-(3',4'-dihydroxyphenyl)-valeric acid | 4.50 | 225.0 / 163.0 | -70 | -10 | -20 | -20 | -2 |  | 225.0 / 181.0 | -70 | -10 | -20 | -35 | -2 | 4,4-Bis-4-hydroxyphenylvaleric acid |
| 4-Hydroxy-5-(4'-hydroxyphenyl)-valeric acid | 6.90 | 209.0 / 147.0 | -70 | -10 | -20 | -15 | -2 |  | 209.0 / 165.0 | -70 | -10 | -20 | -15 | -2 | 4,4-Bis-4-hydroxyphenylvaleric acid |
| 4-Hydroxy-5-(3'-hydroxyphenyl)-valeric acid | 8.30 | 209.0 / 147.0 | -70 | -10 | -20 | -15 | -2 |  | 209.0 / 165.0 | -70 | -10 | -20 | -15 | -2 | 4,4-Bis-4-hydroxyphenylvaleric acid |
| 4-Hydroxy-5-(2'-hydroxyphenyl)-valeric acid | 10.3 | 209.0 / 147.0 | -70 | -10 | -20 | -15 | -2 |  | 209.0 / 165.0 | -70 | -10 | -20 | -15 | -2 | 4,4-Bis-4-hydroxyphenylvaleric acid |
| 4-Hydroxy-5-phenyl-valeric acid | 11.8 | 193.0 / 175.0 | -70 | -10 | -19 | -20 | -2 |  | 193.0 / 149.0 | -70 | -10 | -19 | -20 | -2 | 4,4-Bis-4-hydroxyphenylvaleric acid |
| 5-(3',4'-Dihydroxyphenyl)-valeric acid | 9.80 | 209.0 / 135.0 | -75 | -6 | -20 | -28 | -4 |  | 209.0 / 122.0 | -75 | -6 | -20 | -25 | -3 | 4,4-Bis-4-hydroxyphenylvaleric acid |
| 5-(3'-Hydroxyphenyl)-valeric acid | 11.7 | 193.0 / 165.0 | -70 | -10 | -19 | -20 | -2 |  | 193.0 / 149.0 | -70 | -10 | -19 | -25 | -2 | 4,4-Bis-4-hydroxyphenylvaleric acid |
| 5-(4'-Hydroxyphenyl)-valeric acid | 9.30 | 193.0 / 165.0 | -70 | -10 | -19 | -20 | -2 |  | 193.0 / 149.0 | -70 | -10 | -19 | -25 | -2 | 4,4-Bis-4-hydroxyphenylvaleric acid |
| Phenyl-valeric acid | 13.7 | 177.0 / 133.0 | -55 | -7 | -18 | -25 | -2 |  | 177.0 / 77.0 | -55 | -7 | -18 | -40 | -2 | 4,4-Bis-4-hydroxyphenylvaleric acid |
| 1-(3',4'-Dihydroxyphenyl)-3-(2',4',6'-trihydroxyphenyl)-propan-2-ol | 6.70 | 291.0 / 122.0 | -60 | -10 | -22 | -35 | -2 |  | 291.0 / 247.0 | -60 | -10 | -22 | -25 | -2 | Catechin |
| 1-(4'-Hydroxyphenyl)-3-(2',4',6'-trihydroxyphenyl)-propan-2-ol | 6.00 | 275.0 / 231.0 | -40 | -10 | -22 | -20 | -2 |  | 275.0 / 106.0 | -40 | -10 | -22 | -35 | -2 | Catechin |
| 1-(3'-Hydroxyphenyl)-3-(2',4',6'-trihydroxyphenyl)-propan-2-ol | 8.30 | 275.0 / 231.0 | -40 | -10 | -22 | -20 | -2 |  | 275.0 / 106.0 | -40 | -10 | -22 | -35 | -2 | Catechin |
| 1-(2'-Hydroxyphenyl)-3-(2',4',6'-trihydroxyphenyl)-propan-2-ol | 10.3 | 275.0 / 231.0 | -40 | -10 | -22 | -20 | -2 |  | 275.0 / 106.0 | -40 | -10 | -22 | -35 | -2 | Catechin |
| **Phenyl alcohols^(2)^** | | | | | | | | | | | | | | | |
| Tyrosol | 4.60 | 137.0 / 119.1 | -55 | -9 | -17 | -18 | -2 |  | 137.0 / 105.9 | -55 | -9 | -17 | -18 | -2 | Tyrosol |
| Hydroxytyrosol | 3.50 | 153.0 / 123.0 | -50 | -6 | -17 | -12 | -2 |  | 153.0 / 122.4 | -50 | -6 | -17 | -34 | 0 | Hydroxytyrosol |
| Hydroxytyrosol sulphate | 4.50 | 233.0 / 153.0 | -40 | -8 | -20 | -25 | -2 |  | 233.0 / 123.0 | -40 | -8 | -20 | -40 | -2 | Hydroxytyrosol |
| **Flavanones^(2)^** | | | | | | | | | | | | | | | |
| Naringenin | 13.4 | 271.0 / 151.0 | -40 | -6 | -22 | -30 | -2 |  | 271.0 / 119.0 | -40 | -6 | -22 | -30 | -2 | Naringenin |
| Naringenin pentose | 4.60 | 417.0 / 271.0 | -40 | -6 | -24 | -15 | -2 |  | 417.0 / 271.0 | -40 | -6 | -24 | -15 | -2 | Naringenin |
| Naringenin hexose | 10.4 | 433.0 / 271.0 | -40 | -6 | -24 | -15 | -2 |  | 433.0 / 271.0 | -40 | -6 | -24 | -15 | -2 | Naringenin |
|  |  |  |  |  |  |  |  |  |  |  |  |  |  |  |  |
| **Flavones^(2)^** |  |  |  |  |  |  |  |  |  |  |  |  |  |  |  |
| Apigenin | 13.3 | 268.9 / 117.0 | -85 | -12 | -35 | -46 | -2 |  | 268.9 / 65.1 | -85 | -12 | -35 | -66 | 0 | Apigenin |
| Apigenin-7-glucoside | 10.2 | 431.0 / 268.1 | -100 | -11 | -35 | -38 | -3 |  | 431.0 / 65.2 | -100 | -11 | -35 | -116 | 0 | Apigenin-7-glucoside |
| Luteolin | 12.1 | 285.0 / 132.9 | -80 | -10 | -35 | -46 | -2 |  | 285.0 / 132.1 | -80 | -10 | -35 | -70 | -2 | Luteolin |
| Luteolin-7-glucoside | 9.50 | 447.0 / 285.1 | -100 | -9 | -35 | -34 | -3 |  | 447.0 / 65.0 | -100 | -9 | -35 | -104 | 0 | Luteolin-7-glucoside |
| **Flavonols^(2)^** | | | | | | | | | | | | | | | |
| Isorhamnetin | 13.7 | 314.9 / 79.0 | -90 | -10 | -23 | -20 | -3 |  | 314.9 / 96.9 | -90 | -10 | -23 | -34 | -2 | Isorhamnetin |
| Isorhamnetin-galactoside | 10.3 | 477.0 / 314.0 | -70 | -8 | -29 | -34 | -4 |  | 477.0 / 271.0 | -70 | -8 | -30 | -48 | -4 | Isorhamnetin-glucoside |
| Isorhamnetin-glucoside | 10.4 | 477.0 / 314.0 | -70 | -8 | -29 | -34 | -4 |  | 477.0 / 271.0 | -70 | -8 | -35 | -48 | -4 | Isorhamnetin-glucoside |
| Isorhamnetin-glucuronide | 10.5 | 491.0 / 315.0 | -70 | -9 | -30 | -42 | -4 |  | 491.0 / 151.0 | -70 | -9 | -30 | -42 | -4 | Isorhamnetin-glucoside |
| Isorhamnetin-rutinoside | 10.3 | 623.1 / 314.5 | -90 | -9 | -35 | -40 | -4 |  | 623.1 / 271.0 | -90 | -9 | -35 | -78 | -4 | Isorhamnetin-glucoside |
| Kaempferol | 13.5 | 285.1 / 211.0 | -95 | -9 | -22 | -52 | 0 |  | 285.1 / 255.0 | -95 | -9 | -28 | -58 | -2 | Kaempferol |
| Kaempferol-galactoside | 9.90 | 447.0 / 284.0 | -75 | -11 | -28 | -30 | -4 |  | 447.0 / 254.9 | -75 | -11 | -29 | -46 | -4 | Kaempferol-glucoside |
| Kaempferol-glucoside | 10.2 | 447.0 / 284.0 | -75 | -11 | -28 | -30 | -4 |  | 447.0 / 254.9 | -75 | -11 | -34 | -46 | -4 | Kaempferol-glucoside |
| Kaempferol-glucuronide | 10.2 | 461.0 / 285.0 | -75 | -11 | -29 | -35 | -4 |  | 461.0 / 151.0 | -75 | -11 | -29 | -50 | -4 | Kaempferol-glucoside |
| Kaempferol-rutinoside | 10.2 | 593.0 / 447.0 | -75 | -11 | -34 | -35 | -4 |  | 593.0 / 240.0 | -75 | -11 | -34 | -35 | -4 | Kaempferol-glucoside |
| Miricetin | 10.5 | 316.9 / 151.0 | -80 | -5 | -23 | -30 | -2 |  | 316.9 / 136.9 | -80 | -5 | -23 | -30 | -2 | Miricetin |
| Miricetin-galactoside | 7.80 | 479.1 / 317.0 | -90 | -10 | -29 | -30 | -3 |  | 479.1 / 272.0 | -90 | -10 | -30 | -40 | -3 | Miricetin-galactoside |
| Miricetin-glucoside | 7.90 | 479.1 / 317.0 | -90 | -10 | -29 | -30 | -3 |  | 479.1 / 272.0 | -90 | -10 | -35 | -40 | -3 | Miricetin-galactoside |
| Miricetin-glucuronide | 7.80 | 493.0 / 317.0 | -90 | -6 | -30 | -35 | -4 |  | 493.0 / 179.0 | -90 | -6 | -30 | -35 | -4 | Miricetin-galactoside |
| Miricetin-rutinoside | 7.70 | 625.2 / 479.0 | -90 | -9 | -35 | -35 | -4 |  | 625.2 / 317.0 | -90 | -9 | -35 | -35 | -4 | Miricetin-galactoside |
| Quercetin | 12.2 | 300.7 / 179.0 | -55 | -10 | -23 | -24 | -2 |  | 300.7 / 150.9 | -55 | -10 | -23 | -26 | -2 | Quercetin |
| Quercetin-galactoside | 9.20 | 463.1 / 299.6 | -80 | -9 | -29 | -32 | -3 |  | 463.1 / 270.9 | -80 | -9 | -29 | -48 | -3 | Quercetin-glucoside |
| Quercetin-glucoside | 9.40 | 463.1 / 299.6 | -80 | -9 | -29 | -32 | -3 |  | 463.1 / 270.9 | -80 | -9 | -34 | -48 | -3 | Quercetin-glucoside |
| Quercetin-glucuronide | 9.40 | 477.0 / 301.0 | -80 | -6 | -29 | -30 | -4 |  | 477.0 / 151.0 | -80 | -6 | -29 | -50 | -4 | Quercetin-glucuronide |
| Quercetin-rutinoside | 9.80 | 609.1 / 300.1 | -80 | -10 | -34 | -30 | -4 |  | 609.1 / 271.0 | -80 | -10 | -34 | -50 | -2 | Quercetin-glucoside |
| Laricitrin | 12.3 | 331.0 / 151.0 | -60 | -4 | -24 | -50 | -2 |  | 331.0 / 303.0 | -60 | -4 | -24 | -35 | -2 | Quercetin |
| Laricitrin-galactoside | 9.50 | 493.0 / 330.0 | -60 | -10 | -30 | -50 | -2 |  | 493.0 / 244.0 | -60 | -10 | -24 | -50 | -2 | Syringetin-glucoside |
| Laricitrin-glucoside | 9.60 | 493.0 / 330.0 | -60 | -10 | -30 | -50 | -2 |  | 493.0 / 244.0 | -60 | -10 | -30 | -50 | -2 | Syringetin-glucoside |
| Syringetin | 13.6 | 345.0 / 315.0 | -60 | -4 | -24 | -35 | -2 |  | 345.0 / 330.0 | -60 | -4 | -24 | -30 | -2 | Quercetin |
| Syringetin-galactoside | 10.3 | 507.1 / 344.1 | -60 | -10 | -30 | -50 | -2 |  | 507.1 / 257.8 | -60 | -10 | -28 | -50 | -2 | Syringetin-glucoside |
| Syringetin-glucoside | 10.4 | 507.1 / 344.1 | -60 | -10 | -30 | -50 | -2 |  | 507.1 / 257.8 | -60 | -10 | -28 | -50 | -2 | Syringetin-glucoside |
| Astilbin | 9.90 | 449.0 / 151.0 | -70 | -8 | -28 | -32 | -3 |  | 449.0 / 286.0 | -70 | -8 | -28 | -28 | -3 | Astilbin |
| Neoastilbin | 9.50 | 449.0 / 151.0 | -70 | -8 | -28 | -32 | -2 |  | 449.0 / 285.0 | -70 | -8 | -28 | -28 | -3 | Astilbin |
| **Proanthocyanidins and biological metabolites^(2)^** | | | | | | | | | | | | | | | |
| Catechin | 4.80 | 288.9 / 108.9 | -40 | -10 | -22 | -50 | -2 |  | 288.9 / 122.7 | -40 | -10 | -28 | -50 | -2 | Catechin |
| Catechin sulphate | 4.70 | 369.0 / 289.0 | -50 | -8 | -25 | -25 | -2 |  | 369.0 / 245.0 | -50 | -8 | -25 | -40 | -2 | Catechin |
| Catechin glucuronide | 4.10 | 465.0 / 289.0 | -50 | -8 | -29 | -25 | -2 |  | 465.0 / 245.0 | -50 | -8 | -29 | -40 | -2 | Catechin |
| Methylcatechin sulphate | 7.50 | 383.0 / 303.0 | -50 | -8 | -26 | -25 | -2 |  | 383.0 / 289.0 | -50 | -8 | -26 | -40 | -2 | Catechin |
| Methylcatechin glucuronide | 5.40 | 479.0 / 303.0 | -50 | -8 | -29 | -25 | -2 |  | 479.0 / 289.0 | -50 | -8 | -29 | -40 | -2 | Catechin |
| Epicatechin | 6.60 | 288.9 / 108.9 | -40 | -10 | -22 | -50 | -2 |  | 288.9 / 122.7 | -40 | -10 | -22 | -50 | -2 | Epicatechin |
| Epicatechin sulphate | 7.60 | 369.0 / 289.0 | -50 | -8 | -25 | -25 | -2 |  | 369.0 / 245.0 | -50 | -8 | -25 | -40 | -2 | Epicatechin |
| Epicatechin glucuronide | 4.70 | 465.0 / 289.0 | -50 | -8 | -29 | -25 | -2 |  | 465.0 / 245.0 | -50 | -8 | -29 | -40 | -2 | Epicatechin |
| Methylepicatechin sulphate | 8.40 | 383.0 / 303.0 | -50 | -8 | -26 | -25 | -2 |  | 383.0 / 289.0 | -50 | -8 | -26 | -40 | -2 | Epicatechin |
| Methylepicatechin glucuronide | 6.90 | 479.0 / 303.0 | -50 | -8 | -29 | -25 | -2 |  | 479.0 / 289.0 | -50 | -8 | -29 | -40 | -2 | Epicatechin |
| Epicatechin-gallate | 9.20 | 441.0 / 169.0 | -40 | -10 | -28 | -50 | -2 |  | 441.0 / 289.1 | -40 | -10 | -28 | -50 | -2 | Epicatechin |
| Gallocatechin | 3.10 | 305.0 / 125.0 | -40 | -10 | -23 | -50 | -2 |  | 305.0 / 109.1 | -40 | -10 | -23 | -50 | -2 | Gallocatechin |
| Epigallocatechin | 4.30 | 305.0 / 125.0 | -40 | -10 | -23 | -50 | -2 |  | 305.0 / 109.1 | -40 | -10 | -23 | -50 | -2 | Epicatechin |
| Epigallocatechin-gallate | 5.30 | 457.0 / 169.0 | -40 | -10 | -29 | -50 | -2 |  | 457.0 / 125.0 | -40 | -10 | -29 | -50 | -2 | Epicatechin |
| Procyanidin B1 | 4.20 | 577.1 / 124.9 | -70 | -10 | -26 | -50 | -2 |  | 577.2 / 289.2 | -70 | -10 | -33 | -34 | -4 | Procyanidin B1 |
| Procyanidin B2 | 6.00 | 577.1 / 124.9 | -70 | -10 | -33 | -50 | -2 |  | 577.2 / 289.2 | -70 | -10 | -33 | -34 | -4 | Procyanidin B2 |
| Procyanidin B3 | 7.60 | 577.1 / 124.9 | -70 | -10 | -33 | -50 | -2 |  | 577.2 / 289.2 | -70 | -10 | -33 | -34 | -4 | Procyanidin B1 |
| Procyanidin B4 | 5.60 | 577.1 / 124.9 | -70 | -10 | -33 | -50 | -2 |  | 577.1 / 289.2 | -70 | -10 | -33 | -34 | -4 | Procyanidin B1 |
| Procyanidin B5 | 9.40 | 577.1 / 124.9 | -70 | -10 | -33 | -50 | -2 |  | 577.1 / 289.2 | -70 | -10 | -33 | -34 | -4 | Procyanidin B1 |
| Procyanidin A2 | 9.40 | 575.0 / 285.0 | -90 | -8 | -33 | -36 | -3 |  | 575.0 / 124.9 | -90 | -8 | -33 | -32 | -3 | Procyanidin A2 |
| Procyanidin C1 (trimer) | 7.30 | 865.0 / 125.0 | -85 | -10 | -44 | -70 | 0 |  | 865.0 / 289.0 | -85 | -10 | -44 | -52 | -3 | Procyanidin C1 |
| 5-(3'-Hydroxyphenyl)-ɣ-valerolactone | 9.40 | 191.0 / 147.0 | -75 | -8 | -19 | -17 | -3 |  | 191.0 / 106.0 | -75 | -8 | -19 | -30 | -3 | 5-(3',4'-Dihydroxyphenyl)-δ-valerolactone |
| 5-(4'-Hydroxyphenyl)-ɣ-valerolactone | 8.40 | 191.0 / 147.0 | -75 | -8 | -19 | -17 | -3 |  | 191.0 / 106.0 | -75 | -8 | -19 | -30 | -3 | 5-(3',4'-Dihydroxyphenyl)-δ-valerolactone |
| 5-(3'-Hydroxyphenyl)-ɣ-valerolactone sulphate | 8.40 | 271.0 / 191.0 | -50 | -8 | -22 | -25 | -2 |  | 271.0 / 147.0 | -50 | -8 | -22 | -40 | -2 | 5-(3',4'-Dihydroxyphenyl)-δ-valerolactone |
| 5-(3'-Hydroxyphenyl)-ɣ-valerolactone glucuronide | 7.70 | 367.0 / 191.0 | -50 | -8 | -25 | -25 | -2 |  | 367.0 / 147.0 | -50 | -8 | -25 | -40 | -2 | 5-(3',4'-Dihydroxyphenyl)-δ-valerolactone |
| 5-(3',4'-Dihydroxyphenyl)-ɣ-valerolactone | 6.70 | 207.0 / 163.0 | -75 | -8 | -19 | -20 | -3 |  | 207.0 / 122.0 | -75 | -8 | -19 | -25 | -3 | 5-(3',4'-Dihydroxyphenyl)-δ-valerolactone |
| 5-(3',4'-Dihydroxyphenyl)-δ-valerolactone | 6.50 | 207.0 / 85.0 | -75 | -6 | -19 | -28 | -4 |  | 207.0 / 121.0 | -75 | -6 | -19 | -25 | -3 | 5-(3',4'-Dihydroxyphenyl)-δ-valerolactone |
| 5-(3',4'-Dihydroxyphenyl)-ɣ-valerolactone sulphate | 7.40 | 287.0 / 207.0 | -50 | -8 | -22 | -25 | -2 |  | 287.0 / 163.0 | -50 | -8 | -22 | -40 | -2 | 5-(3',4'-Dihydroxyphenyl)-δ-valerolactone |
| 5-(3',4'-Dihydroxyphenyl)-ɣ-valerolactone glucuronide | 6.50 | 383.0 / 207.0 | -50 | -8 | -26 | -25 | -2 |  | 383.0 / 163.0 | -50 | -8 | -26 | -40 | -2 | 5-(3',4'-Dihydroxyphenyl)-δ-valerolactone |
| 5-(3',4'-Dihydroxyphenyl)-ɣ-valerolactone sulphate glucuronide | 4.60 | 463.0 / 287.0 | -50 | -8 | -29 | -50 | -2 |  | 463.0 / 163.0 | -50 | -8 | -29 | -50 | -2 | 5-(3',4'-Dihydroxyphenyl)-δ-valerolactone |
| **Dihydrochalcones^(2)^** | | | | | | | | | | | | | | | |
| Phloridzin | 10.9 | 435.4 / 273.3 | -40 | -10 | -28 | -40 | -2 |  | 435.4 / 167.0 | -40 | -10 | -28 | -50 | -2 | Phloridzin |
| **Stilbenes^(2)^** | | | | | | | | | | | | | | | |
| *trans*-Resveratrol | 10.9 | 227.0 / 142.9 | -30 | -10 | -20 | -30 | -2 |  | 227.0 / 185.2 | -30 | -10 | -20 | -18 | -2 | *trans*-Resveratrol |
| *cis*-Resveratrol | 12.6 | 227.0 / 142.9 | -40 | -10 | -20 | -30 | -2 |  | 227.0 / 185.2 | -40 | -10 | -20 | -18 | -2 | *trans*-Resveratrol |
| *trans*-Resveratrol glucoside | 8.80 | 389.0 / 227.1 | -40 | -10 | -26 | -30 | -2 |  | 389.0 / 184.9 | -40 | -10 | -26 | -50 | -2 | *trans*-Resveratrol glucoside |
| *cis-*Resveratrol glucoside | 10.8 | 389.0 / 227.1 | -40 | -10 | -26 | -30 | -2 |  | 389.0 / 184.9 | -40 | -10 | -26 | -50 | -2 | *trans*-Resveratrol glucoside |
| Resveratrol sulphate | 11.3 | 307.0 / 227.0 | -40 | -9 | -23 | -30 | -4 |  | 307.0 / 185.0 | -40 | -9 | -23 | -40 | -4 | *trans*-Resveratrol |
| Resveratrol glucuronide | 10.6 | 403.0 / 227.0 | -70 | -9 | -27 | -30 | -4 |  | 403.0 / 185.0 | -70 | -9 | -27 | -40 | -4 | *trans*-Resveratrol |
| *trans*-Resveratrol glucuronide sulphate | 8.90 | 483.0 / 227.0 | -40 | -9 | -30 | -40 | -4 |  | 483.0 / 142.9 | -40 | -9 | -30 | -40 | -4 | *trans*-Resveratrol |
| Dihydroresveratrol | 10.9 | 229.0 / 123.0 | -30 | -10 | -20 | -20 | -2 |  | 229.0 / 81.0 | -30 | -10 | -20 | -35 | -2 | *trans*-Resveratrol |
| Dihydroresveratrol sulphate | 10.5 | 309.3 / 229.0 | -40 | -9 | -23 | -30 | -4 |  | 309.3 / 123.0 | -40 | -9 | -23 | -40 | -4 | *trans*-Resveratrol |
| Dihydroresveratrol glucuronide | 9.80 | 405.4 / 229.0 | -50 | -8 | -27 | -30 | -4 |  | 405.4 / 123.0 | -50 | -8 | -27 | -50 | -4 | *trans*-Resveratrol |
| Dihydroresveratrol glucuronide sulphate | 9.90 | 485.0 / 229.0 | -40 | -9 | -30 | -40 | -4 |  | 485.0 / 123.0 | -40 | -9 | -30 | -40 | -4 | *trans*-Resveratrol |
| ԑ-Viniferin | 11.5 | 453.0 / 359.0 | -40 | -10 | -28 | -50 | -2 |  | 453.0 / 225.0 | -40 | -10 | -28 | -50 | -2 | *trans*-Resveratrol |
| ω-Viniferin | 13.5 | 453.0 / 225.0 | -40 | -10 | -28 | -50 | -2 |  | 453.0 / 347.0 | -40 | -10 | -28 | -50 | -2 | *trans*-Resveratrol |
| *trans*-Piceatannol | 9.50 | 243.0 / 159.1 | -40 | -10 | -21 | -50 | -2 |  | 243.0 / 200.9 | -40 | -10 | -20 | -50 | -2 | *trans*-Resveratrol |
| *cis*-Piceatannol | 11.2 | 243.0 / 159.1 | -40 | -10 | -21 | -50 | -2 |  | 243.0 / 200.9 | -40 | -10 | -21 | -50 | -2 | *trans*-Resveratrol |
| *trans*-Astringin | 7.20 | 405.0 / 243.0 | -40 | -10 | -27 | -50 | -2 |  | 405.0 / 200.9 | -40 | -10 | -27 | -50 | -2 | *trans*-Resveratrol glucoside |
| *cis*-Astringin | 9.70 | 405.0 / 243.0 | -40 | -10 | -27 | -50 | -2 |  | 405.0 / 200.9 | -40 | -10 | -27 | -50 | -2 | *trans*-Resveratrol glucoside |
| **Lignans and metabolites^(2)^** | | | | | | | | | | | | | | | |
| Matairesinol | 13.5 | 357.2 / 83.1 | -70 | -10 | -25 | -30 | -2 |  | 357.2 / 122.1 | -70 | -10 | -31 | -40 | -2 | Matairesinol |
| Secoisolaricicresinol | 11.0 | 361.1 / 122.0 | -70 | -10 | -25 | -40 | -2 |  | 361.1 / 165.0 | -70 | -10 | -25 | -30 | -2 | Secoisolaricicresinol |
| Matai/Pino resinol-glucoside | 11.0 | 519.0 / 357.0 | -70 | -10 | -31 | -40 | -2 |  | 519.0 / 357.0 | -70 | -10 | -31 | -40 | -2 | Matairesinol |
| Secoisolariciresinol-glucoside | 10.0 | 523.2 / 361.0 | -70 | -10 | -31 | -40 | -2 |  | 523.2 / 361.0 | -70 | -10 | -31 | -40 | -2 | Secoisolaricicresinol |
| Isolariciresinol-glucoside | 7.30 | 521.0 / 359.0 | -70 | -10 | -31 | -40 | -2 |  | 521.0 / 344.0 | -70 | -10 | -31 | -40 | -2 | Secoisolaricicresinol |
| Lariciresinol-glucoside | 10.2 | 593.0 / 447.0 | -75 | -11 | -31 | -35 | -4 |  | 593.0 / 240.0 | -75 | -11 | -31 | -35 | -4 | Secoisolaricicresinol |
| Enterodiol | 10.4 | 301.1 / 253.0 | -80 | -8 | -23 | -20 | -2 |  | 301.1 / 271.0 | -80 | -8 | -23 | -30 | -2 | Secoisolariciresinol |
| Enterodiol sulphate | 10.1 | 381.1 / 301.1 | -80 | -9 | -26 | -40 | -4 |  | 381.1 / 253.0 | -80 | -9 | -26 | -40 | -4 | Secoisolariciresinol |
| Enterodiol glucuronide | 10.3 | 477.1 / 253.0 | -80 | -9 | -30 | -40 | -4 |  | 477.1 / 271.0 | -80 | -9 | -30 | -40 | -4 | Secoisolariciresinol |
| Enterodiol glucuronide sulphate | 9.80 | 557.1 / 253.0 | -80 | -9 | -32 | -40 | -4 |  | 557.1 / 271.0 | -80 | -9 | -32 | -40 | -4 | Secoisolariciresinol |
| Enterolactone | 12.1 | 297.1 / 189.0 | -70 | -8 | -23 | -30 | -2 |  | 297.1 / 253.0 | -70 | -8 | -23 | -30 | -2 | Matairesinol |
| Enterolactone sulphate | 10.6 | 377.1 / 297.1 | -80 | -9 | -26 | -40 | -4 |  | 377.1 / 189.0 | -80 | -9 | -26 | -40 | -4 | Matairesinol |
| Enterolactone glucuronide | 11.6 | 473.1 / 189.0 | -80 | -9 | -30 | -40 | -4 |  | 473.1 / 253.0 | -80 | -9 | -30 | -40 | -4 | Matairesinol |
| Enterolactone glucuronide sulphate | 10.5 | 553.1 / 189.0 | -80 | -9 | -32 | -40 | -4 |  | 553.1 / 253.0 | -80 | -9 | -32 | -40 | -4 | Matairesinol |
| **Catechols, pyrogallols and phloroglucinols ^(2)^** | | | | | | | | | | | | | | | |
| Phloroglucinol | 1.70 | 125.0 / 57.0 | -55 | -6 | -16 | -18 | 0 |  | 125.0 / 83.0 | -55 | -6 | -16 | -22 | 0 | Phloroglucinol |
| Catechol | 3.80 | 109.0 / 91.0 | -60 | -10 | -16 | -35 | -1 |  | 109.0 / 81.0 | -60 | -10 | -16 | -24 | 0 | Catechol |
| Pyrogallol | 2.10 | 125.0 / 79.0 | -45 | -10 | -16 | -27 | -2 |  | 125.0 / 81.0 | -45 | -10 | -16 | -26 | -2 | Pyrogallol |
| **Tannins and metabolites^(2)^** | | | | | | | | | | | | | | | |
| Urolithin A | 11.4 | 226.9 / 154.1 | -85 | -9 | -20 | -46 | -4 |  | 226.9 / 197.9 | -85 | -9 | -20 | -30 | -8 | Urolithin A |
| Urolithin B | 13.8 | 210.9 / 167.0 | -85 | -4 | -19 | -26 | -2 |  | 210.9 / 139.0 | -85 | -4 | -19 | -26 | -4 | Urolithin B |
| Urolithin C | 9.90 | 243.0 / 187.0 | -85 | -7 | -21 | -35 | -2 |  | 243.0 / 215.0 | -85 | -7 | -21 | -40 | -2 | Urolithin A |
| Urolithin B glucuronide | 12.9 | 387.0 / 211.0 | -95 | -9 | -26 | -40 | -4 |  | 387.0 / 167.0 | -95 | -9 | -26 | -40 | -4 | Urolithin B |

RT: Retention Time; DP: Declustering Potential; EP: Entrance Potential; CEP: Collision Cell Entrance Potential; CE: Collision Energy; CXP: Collision Cell Exit Potential

^(1)^ Positive ionization mode

^(2)^ Negative ionization mode
