# Supplementary material for: Development of a Wine By‐Product‐Based Beverage and Study of Its Potential to Postprandial Glycemia Regulation in Healthy Individuals: A Proof of Concept Study
Source: Mol Nutr Food Res. 2025 May 27;69(14):e70128. doi: 10.1002/mnfr.70128 (PMC12280843; doi:10.1002/mnfr.70128)
Supplement: Supplementary file 4 — Supporting information [file MNFR-69-e70128-s001.docx]

**Supplemental Table S3**. Calculated Intensity (%), Frequency (%) and Global Media (%) of the different attributes included in the testing sheet of each WBB beverage formulation (1-4).

| Attribute | Formulation 1 | | |  | Formulation 2 | | |  | Formulation 3 | | |  | Formulation 4 | | |
| --- | --- | --- | --- | --- | --- | --- | --- | --- | --- | --- | --- | --- | --- | --- | --- |
|  | I (%) | F (%) | GM (%) |  | I (%) | F (%) | GM (%) |  | I (%) | F (%) | GM (%) |  | I (%) | F (%) | GM (%) |
| *Appearance* |  |  |  |  |  |  |  |  |  |  |  |  |  |  |  |
| Brightness | 47.7 | 100 | 69.1 |  | 62.0 | 100 | 78.8 |  | 53.7 | 100 | 73.3 |  | 70.4 | 100 | 83.9 |
| Pink-violet | 75.5 | 100 | 86.9 |  | 77.7 | 100 | 88.2 |  | 61.6 | 100 | 78.5 |  | 78.1 | 100 | 88.3 |
| Homogeneity | 59.9 | 100 | 77.4 |  | 63.8 | 100 | 79.9 |  | 40.3 | 100 | 63.5 |  | 62.1 | 100 | 78.8 |
| Density | 58.4 | 100 | 76.4 |  | 66.5 | 100 | 81.5 |  | 49.8 | 100 | 70.6 |  | 68.8 | 100 | 82.9 |
| Sandy | 75.1 | 100 | 86.6 |  | 73.3 | 100 | 85.6 |  | 61.5 | 100 | 78.5 |  | 68.1 | 100 | 82.5 |
| *Odour* |  |  |  |  |  |  |  |  |  |  |  |  |  |  |  |
| Pressed grape skin | 44.6 | 100 | 66.8 |  | 58.1 | 100 | 76.2 |  | 46.5 | 100 | 68.2 |  | 52.0 | 91.67 | 69.0 |
| Grape stem | 35.4 | 83.3 | 54.3 |  | 36.7 | 91.7 | 58.0 |  | 30.1 | 83.3 | 50.1 |  | 30.7 | 83.33 | 50.5 |
| Herbaceous | 39.5 | 91.7 | 60.2 |  | 28.2 | 83.3 | 48.5 |  | 28.0 | 91.7 | 50.7 |  | 31.2 | 91.67 | 53.4 |
| Acidic | 26.3 | 83.3 | 46.7 |  | 29.1 | 83.3 | 49.2 |  | 26.1 | 91.7 | 48.9 |  | 26.9 | 100 | 51.8 |
| Red berries | 36.7 | 83.3 | 55.3 |  | 29.6 | 83.3 | 49.7 |  | 28.5 | 91.7 | 51.1 |  | 35.9 | 91.67 | 57.3 |
| Unripe fruit | 35.4 | 83.3 | 54.3 |  | 26.0 | 75.0 | 46.6 |  | 28.9 | 91.7 | 51.5 |  | 25.1 | 91.67 | 47.9 |
| Citric | 18.5 | 83.3 | 39.3 |  | 14.9 | 75.0 | 33.4 |  | 21.5 | 83.3 | 42.3 |  | 19.5 | 100 | 44.1 |
| Floral | 26.7 | 83.3 | 47.2 |  | 32.2 | 91.7 | 54.3 |  | 30.6 | 91.7 | 52.9 |  | 43.7 | 100 | 66.0 |
| Overall intensity | 57.7 | 100 | 75.9 |  | 56.7 | 100 | 75.3 |  | 48.1 | 100 | 69.4 |  | 66.4 | 100 | 81.5 |
| Overall quality | 52.6 | 100 | 72.5 |  | 58.8 | 100 | 76.7 |  | 42.0 | 100 | 64.8 |  | 63.5 | 100 | 79.6 |
| *Taste* |  |  |  |  |  |  |  |  |  |  |  |  |  |  |  |
| Overall quality | 45.0 | 100 | 67.1 |  | 49.0 | 91.67 | 67.0 |  | 44.6 | 100 | 66.8 |  | 49.1 | 100 | 70.0 |
| Sweet | 22.8 | 83.3 | 43.6 |  | 17.5 | 75.0 | 36.2 |  | 18.4 | 100 | 42.9 |  | 18.3 | 66.7 | 34.9 |
| Salty | 12.3 | 66.7 | 28.6 |  | 16.9 | 58.3 | 31.4 |  | 21.7 | 58.3 | 35.6 |  | 15.3 | 66.7 | 31.9 |
| Acidic | 53.0 | 91.7 | 69.7 |  | 66.2 | 100 | 81.4 |  | 34.5 | 91.7 | 56.2 |  | 618 | 100 | 78.5 |
| Bitter | 29.0 | 83.3 | 49.1 |  | 33.1 | 83.3 | 52.5 |  | 24.6 | 83.3 | 45.3 |  | 32.0 | 75.0 | 48.9 |
| *Mouth feel (texture)* |  |  |  |  |  |  |  |  |  |  |  |  |  |  |  |
| Astringence | 36.0 | 83.3 | 54.8 |  | 42.6 | 91.7 | 62.5 |  | 30.7 | 83.3 | 50.6 |  | 45.1 | 91.7 | 64.2 |
| Body | 54.2 | 100 | 73.6 |  | 673 | 100 | 82.0 |  | 42.2 | 100 | 65.0 |  | 58.6 | 91.7 | 73.3 |
| Persistency | 48.8 | 100 | 69.8 |  | 66.2 | 100 | 81.4 |  | 39.8 | 100 | 63.1 |  | 62.4 | 100 | 79.0 |
| Graininess | 31.9 | 100 | 56.5 |  | 37.8 | 100 | 61.5 |  | 31.2 | 91.7 | 53.5 |  | 48.3 | 91.7 | 66.5 |
| Sundy | 52.3 | 100 | 72.3 |  | 70.8 | 100 | 84.1 |  | 48.7 | 100 | 69.8 |  | 28.8 | 100 | 113 |
| *Global quality* | 50.0 | 100 | 70.7 |  | 50.4 | 100 | 71.0 |  | 46.6 | 100 | 68.3 |  | 53.8 | 100 | 73.3 |
